# Metabolomic and lipidomic signatures of aging in cats reveal shifts in energy metabolism and inflammation: an 11-year longitudinal study

**DOI:** 10.3389/fvets.2026.1953163

**Published:** 2026-10-21

**Authors:** Olle Hartvigsson, Alastair B. Ross, Alexandra Moesta, Axel Heiser, Erin Lee, Christina F. Butowski, David G. Thomas, Jeremy Laxalde, Emma N. Bermingham, Delphine Moniot

**Affiliations:** 1 New Zealand Institute for Bioeconomy Science Limited, Lincoln, New Zealand; 2 Royal Canin Research Centre, Aimargues, France; 3 New Zealand Institute for Bioeconomy Science Limited, Palmerston North, New Zealand; 4 School of Agriculture and Environment, Massey University, Palmerston North, New Zealand; 5 EmBer Pet Nutrition, Palmerston North, New Zealand

**Keywords:** aging, feline, inflammaging, lipidomics, longitudinal study, metabolomics

## Abstract

**Background:**

Aging encompasses multiple biochemical changes, some of which may be associated with the development of age-related diseases. The dynamics of aging in relation to metabolism in cats has not been established and understanding this could identify targets for early intervention.

**Objectives:**

The aim of this longitudinal study was to identify changes in the plasma and fecal metabolome and lipidome of cats from 1 to 11 years of age.

**Methods:**

Twenty-one cats from a research colony had plasma and fecal samples collected yearly between one and 11 years of age. Clinical biomarkers were measured in serum, while plasma and fecal metabolomics and lipidomics were measured using mass spectrometry. Data were analyzed using repeated measures-ANOVA Simultaneous Component Analysis.

**Results:**

Two metabolic trajectories were observed: one linear, suggestive of progressive accumulation of metabolic alterations with aging, and a U-shape, suggestive of more complex metabolic changes over time. Three themes emerged: inflammaging (linear decrease of plasma albumin:globulin ratio, increase in fecal inflammatory mediators), mitochondrial dysfunction (linear increase of plasma long-chain phosphatidylcholines, U-shape for plasma hydroxy-butyric acids and lactic acid, inverse U-shape for fecal coenzyme Q), and a possible link to an aging gut microbiome (tryptophan pathway with linear increase of plasma kynurenine versus decrease of fecal anthranilic acids).

**Conclusion:**

Metabolic changes in plasma and feces support the inflammaging hypothesis for cats, alongside mitochondrial dysfunction, and possible changes to gut microbial metabolism. Clear metabolic shifts between 6 and 8 years of age suggest that early intervention could help to support a healthy aging trajectory in cats.

## Graphical abstract

**Figure:**
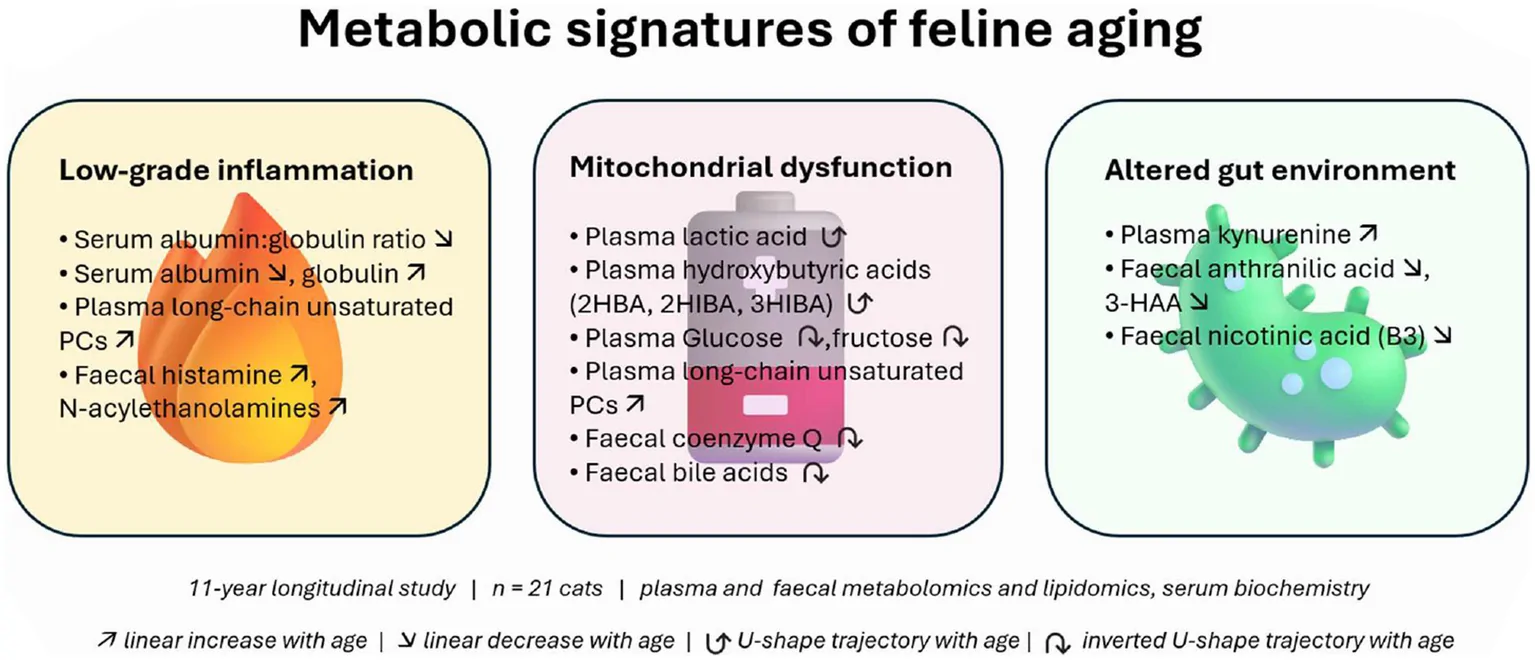

## 1 Introduction

Lifespan of companion animals has increased thanks to advances in veterinary care and nutrition, which comes with a parallel need to improve healthspan (1, 2). Companion animals share the same environment and lifestyle as humans, and are therefore increasingly recognized as a model for geroscience, providing translational insights relevant to the One Health concept (3–5). This involves a better understanding of age-related changes in physiology, particularly those involving metabolic processes (6), and omics sciences (genomics, transcriptomics, proteomics, metabolomics) are useful assets to better elucidate the processes involved (7). While several initiatives in this goal have been reported in dogs (3–5), studies investigating age-related changes in cats are lacking.

Aging biology is intimately associated with dysregulated metabolism, one of the hallmarks of aging (2, 7, 8). Aging encompasses multiple biochemical changes which may start several years before clinical changes. In cats, age-associated diseases include diabetes mellitus (9), hyperthyroidism (10), renal disease (11), and cognitive dysfunction (12). Other physiological processes such as sarcopenia are hallmarks of aging in cats (13). Earlier identification of associated metabolic changes may allow earlier medical, dietary or lifestyle interventions, eventually leading to improved healthspan and lifespan.

While the human literature includes several longitudinal and/or large scale metabolomic studies on the effect of age (14), the veterinary literature in this field is limited, with only a few studies on dogs (6). In cats, only one prospective longitudinal cohort study, with over 200 cats at ages seven-ten years at inclusion (CatPAWS, Cat Prospective Aging and Welfare Study) (10) has been published (15), with changes in the serum metabolome and lipidome in a subset of 163 cats measured yearly or six-monthly at up to five timepoints. Amongst the effects of increasing age on serum metabolites, they observed alterations to serum amino acid concentrations, alterations in pyruvate and glycolysis pathways, and an increase in acetyl-glycoproteins, a measure of systemic inflammation. This suggests changes to energy and protein metabolism in middle aged and older cats but does not track how metabolism changes across the whole feline lifespan. As blood metabolite levels are under tight homeostatic control, there is value in adding analysis of other samples for a better understanding of overall metabolism. Feces represent a readily accessible sample, and fecal metabolomics analyses can help to provide insight into possible impacts of aging on the feline gut microbiome (16, 17).

To understand the metabolic trajectory of the metabolome and lipidome during the lifespan of cats, we have conducted an 11-year longitudinal study on plasma and fecal metabolomes and lipidomes in 21 colony cats, with yearly sampling from the age of 1 year.

## 2 Materials and methods

### 2.1 Animals

All cats in this study (*n* = 21) were adult domestic short hair (*Felis catus*), housed at the Centre for Feline Nutrition (Massey University, Palmerston North, longitude 175°38′E, latitude 40°22’S), an established multigenerational research colony. Cats were cared for according to the New Zealand Animal Welfare (Companion Cats) Code of Welfare (2007), and were housed in purpose-built covered outdoor enclosures (140 × 440 × 240 cm) in groups of seven to ten animals, exposed to natural photoperiods.

Annual collections of fecal and plasma samples were conducted throughout the cats’ lifespan. Cats were included in this prospective, longitudinal study if they had consecutive plasma and fecal samples available in the biobank (between 2011 and 2021) at time of analysis and were not enrolled in another research study at the time of sampling. All sampling was carried out at end of each November, allowing standardization of age at sampling and weight variables.

Of the 21 cats; nine were male and 12 female;19 were neutered and two entire (both female). For *n* = 11 cats, basal diet was a commercially available complete and balanced retorted diet (Chef, Kraft Heinz, New Zealand) which was fed *ad libitum* throughout the sampling period. In addition, within their lifetime these cats were involved in other nutritional research studies, which included diet changes. The other *n* = 10 cats were used for different prospective studies previously reported (18–21) and fed either an extruded (*n* = 4) or a retorted diet (*n* = 6) *ad libitum* throughout the sampling period. Water was available *ad libitum*. Cats were weighed weekly throughout their life, and at the time of selection for this study were all considered healthy. At the time of data analysis, all cats remained free of apparent age-related diseases.

All cats received routine core vaccinations annually in accordance with current feline vaccination guidelines and standard veterinary practice in New Zealand, including vaccination against *Bordetella bronchiseptica* (Canvac, Zoetis).

Collection of the samples for this study was approved by the Massey University Animal Ethics Committee (MUAEC 09/63; 10/108; 12/12; 15/08; 16/24; 17/107; 18/09; 18/16). The number of cats per year of age is tabulated in Supplementary Table 1.

### 2.2 Blood sample collection

Food was withdrawn on the night before each sample collection, resulting in an estimated 12–18 h fasting, depending on the time of last meal taken by each cat, and their order in the batch of cats to be sampled. Samples were collected via jugular venipuncture, following application of local anesthetic gel, into appropriate collection tubes. Samples were stored at −80 °C until they were analyzed. Samples were submitted to a commercial veterinary diagnostic laboratory (Awanui Veterinary, Palmerston North, New Zealand; accredited to NZS/ISO 15189:2022) for biochemical and hematological assays, and processed in-house for metabolomics and lipidomics (detailed below).

### 2.3 Fecal sample collection

Cats were housed individually for a period of up to 24 h until a fresh fecal sample was collected from each animal within 15 min of excretion. Samples were snap-frozen in liquid nitrogen and stored at −80 °C until analysis. Following sample collection, cats returned to group housing.

### 2.4 Serum biochemistry

Serum biochemistry and hormone assays were performed by a commercial veterinary diagnostic laboratory (Awanui Veterinary, Palmerston North, New Zealand). Their standard feline biochemistry panel comprised albumin, globulin, total protein, albumin:globulin ratio, alkaline phosphatase, alanine aminotransferase, aspartate aminotransferase, bilirubin, calcium, cholesterol, chloride, creatine kinase, creatinine, potassium, sodium, sodium:potassium ratio, phosphate, and urea. Serum amyloid A (SAA), cortisol and progesterone were also measured. All analyses were carried out on the laboratory’s routine automated analyzers under its accredited quality-management system (IANZ accreditation to ISO/IEC 17025:2017 for biological testing), and results were interpreted against the laboratory’s validated feline reference intervals.

### 2.5 Immune measures

An indirect ELISA was performed to quantify vaccine specific IgG antibodies (VET 1:80 and VET titre) in feline serum samples. Microtitre plates were coated overnight at 4 °C with a 1:20 dilution of *Bordetella bronchiseptica* antigen (Canvac, Zoetis) in carbonate–bicarbonate buffer, followed by blocking with 10% casein in PBST for 1 h at room temperature. Serum samples were diluted 1:80 and subjected to ten two-fold serial dilutions across the plate (up to 1:81,920); a background control (no serum) was included. After incubation, plates were washed and bound antibodies were detected using HRP-conjugated goat anti-cat IgG (Bio-Rad AA126, 1:10,000). TMB substrate was added, reactions were stopped with 0.5 M H₂SO₄, and absorbance was measured at 450 nm. Background control values were averaged, and the antibody titre for each sample was defined as the highest dilution that produced a signal exceeding the mean background control value +2 standard deviations.

### 2.6 Metabolomics and lipidomics

Plasma and fecal samples were randomized, extracted and analyzed as outlined below.

#### 2.6.1 Sample randomization

The order of samples for metabolomics and lipidomics analyses was randomized using two-step randomization. Cats were randomized into different analytical batches, and samples from the same cat run together and further randomized by year.

#### 2.6.2 Sample extraction

##### 2.6.2.1 Plasma

###### 2.6.2.1.1 Metabolomics

Ten μL of plasma was pipetted into a 2 mL microcentrifuge tube, and 20 μL of in-house internal standard mix added, along with 100 μL of methanol:water (9:1, v/v). Tubes were shaken for 5 min at 30 Hz in a bead shaker (TissueLyser II, Qiagen). Tubes were then placed at −20 °C for 30 min and then centrifuged (10 min, 14 00 x g at 4 °C). Eighty-five μL of the supernatant was transferred to an amber chromatography vial with insert. Remaining extract was pooled to make a quality control sample. Samples were dried in a vacuum centrifuge concentrator (Christ RVC 2–18 CD plus, Martin Christ) and derivatized by methoxymation and silylation.

###### 2.6.2.1.2 Lipidomics

Plasma samples were extracted for lipidomics based on the method of (22). Ten μL of plasma was pipetted into a 2 mL microcentrifuge tube, and 5 μL of internal standard mix (Splash Mix, Avanti Polar Lipids, Alabaster, Alabama, United States) and 95 μL of butanol:methanol (1:1 v/v) with 5 mM ammonium formate, added. The sample was shaken for 5 min at 30 Hz in a bead shaker (TissueLyser II, Qiagen). The samples were then sonicated for 60 min at 20 °C before centrifugation (14,000 x g for 10 min at 20 °C, Heraeus Megafuge 8R, Thermo Fisher). 80 μL of supernatant was aliquoted into an amber chromatography vial with 250 μL flat bottom insert, and 20 μL supernatant transferred to a tube for making a pooled quality control sample.

##### 2.6.2.2 Feces (metabolomics and lipidomics)

Fifty mg of fecal material was weighed into a microcentrifuge tube, and a metal bead added. To the same tube, 20 μL of in-house internal standard mix and 5 μL of lipid internal standard mix (Splash Mix, Avanti Polar Lipids, Alabaster, Alabama, United States) were added, along with 400 μL of 75% methanol. Samples were mixed using a bead mill (TissueLyser II, Qiagen, Hilden, Germany), shaking for 1 min at 30 Hz. Samples were sonicated for 2 min and then put at −20 °C for 10 min. 1 mL of methyl-tert-butyl ether (MTBE) was added and the samples vortexed for 1 h at 450 rpm in a multitube vortex mixer (iSwix). 250 μL of Liquid Chromatography Mass Spectrometry (LC-MS) grade water was added to each tube, and the samples vortexed at 2500 rpm for 1 min. Phase separation was left to occur for 10 min and then the samples centrifuged at 14000 x g for 25 min at 4 °C. Five hundred μL of the upper polar phase was removed for lipidomics analysis. An additional 300 μL of LC-MS grade water was added, and samples were further centrifuged for 20 min at 14000 x g at 4 °C. The remaining polar phase was transferred into a new microcentrifuge tube and centrifuged for 20 min at 14000 x g at 4 °C. Two hundred μL of the polar extract was transferred to an amber chromatography vial with insert and dried in a vacuum centrifuge concentrator (Christ RVC 2–18 CD plus, Martin Christ) before derivatization by methoxymation and silylation.

#### 2.6.3 GC-MS/MS metabolomics

Gas Chromatography Mass Spectrometry (GC-MS/MS) analyses were carried out on a GCMS TQ8040 instrument (Shimadzu) using the SmartMetabolites multiple reaction monitoring (MRM) method. In brief, this targeted metabolomics method includes 450 MRM transitions, covering approximately 400 individual metabolites (several metabolites have two chromatographic peaks). Analysis conditions were: injector temperature 250 °C, interface temperature 280 °C, and MS source temperature 200 °C. The chromatography was carried out on a BPX5 column (30 m x 0.25 mm, 0.25 μm film thickness) with the following temperature gradient: start 60 °C, 2 min 60 °C, 20 min 330 °C, 23 min 330 °C. Two μL of derivatized sample was injected in split injection mode with a ratio of 1:30, and carrier gas linear velocity was held constant at 40 cm/s. Peaks were identified based on ratio of quantifier and qualifier ion and retention index.

#### 2.6.4 LC-MS lipidomics

Lipidomics analyses were carried out using LC-qToF-MS (LCMS 9030, Shimadzu, Kyoto, Japan) in data independent analysis mode. Samples (2 μL) were injected onto a C18 column (Acquity CSH-C18, 100 × 2.1 mm, 1.7 μm). Solvent A was water/acetonitrile/isopropanol (50/30/20 v/v) containing 20 mM ammonium formate, and solvent B was water/acetonitrile/isopropanol (1/9/90 v/v) containing 20 mM ammonium formate. Solvents were LC-MS grade from ThermoFisher (Auckland, New Zealand). The gradient program was as follows: starting, 10% B, 2.7 min, 45% B; 2.8 min, 53% B; 9 min, 65% B; 9.1 min, 89% B; 11 min, 92% B; 11.1 min, 100% B; 13.9 min, 100% B; 14 min, 10% B; 17 min, 10% B. The flow rate was 0.4 mL/min. The column oven was set to 60 °C and autosampler set to 20 °C. The mass spectrometer was run in data independent analysis mode with an electrospray ionization source. This was set up as follows: scanning data between m/z 250 and 1,300 in ToF mode with an event time of 0.03 s, and 41 windows of m/z 20 from m/z 300 to 1,100, in MS/MS mode with a collision energy of 23 and a collision energy spread of 6, and event time of 0.02 s. Analyses were carried out in positive ionization mode.

LC-MS data were processed using MS DIAL (23), including peak deconvolution, database-based identification, and retention time and mass alignment. Data were normalized using LOWESS normalization based on a quality control sample between every ten sample injections.

Lipidomics identifications were refined based on modeling likely retention times based on lipid standards. Lipid identifications that fell outside a +/− 10% retention time range were considered to be incorrectly identified.

### 2.7 Data preprocessing

All data processing was performed in R version 4.4.0 (24).

Outliers due to analytical errors were identified by comparing the total intensity to the blank injections and subsequently removed, if too low (signal to noise <5). Features were filtered and removed if they were not present in at least 50% of injections. Further features were removed, if they were only present in certain batches. Inter- and intra-batch intensity correction was performed on Quality Control (QC) samples using LOESS based correction (span parameter = 0.75). Relative standard deviation (RSD) in QC samples was used as a feature quality metric and features with RSD_QC_ > 0.3 were removed.

Box-Cox transformations for λ = −1, −0.5, 0, 0.5 and 1 were investigated for all metabolomic features and normality assessment was carried out using the Shapiro–Wilk test to evaluate which transformation was most suitable for the data.

### 2.8 Data analysis

Repeated Measures – ANOVA Simultaneous Component Analysis^+^ (RM-ASCA^+^) (25), was used to identify multivariate time-dependent patterns, using the Assorted Linear functions for ASCA (ALASCA) R-package (26). Briefly, this method combines linear mixed models in combination with principal component analysis (PCA) to isolate variation related to selected dependent variables (age) and covariates (sex, diet and weight) while removing unrelated variance. PCA is then applied to the individual fixed effects to facilitate interpretation of one-dimensional patterns, in this case, age. Equation 1 was used in RM-ASCA, with age as a categorical factor variable.


value~Age+Sex+Diet+Weight+(1∣ID) (1)

Confidence intervals were computed using bootstrapping, all parameter settings for RM-ASCA^+^ are available in Supplementary Table 2.

Variables of interest were selected based on the loadings in the principal components from the RM-ASCA^+^ models. RM-ASCA^+^ was further used to extract the fixed effects of the 10 individual variables with highest absolute values on the loadings corresponding to principal component 1. Linear mixed models with restricted maximum likelihood estimation (lme4 R package) (27) were used to further investigate these metabolites of interest. Linear mixed models were evaluated based on Q-Q plots, histograms of residuals and heteroscedasticity. In cases where the residuals showed clear outliers, the outliers were removed and analysis re-executed (Supplementary Figure 1). The formula used in the linear mixed models for individual metabolites were determined based on the fixed effects obtained through ALASCA, Equation 2 was used if the fixed effect showed a linear trend, while Equation 3 was used if the fixed effect showed a quadratic pattern. If pattern was unclear, both formulas were applied and model with lower Akaike Information Criterion (AIC) value was chosen. *p* values were extracted using the lmertest R package (28) and adjusted using Benjamini-Hochberg false discovery rate (FDR) correction. Variables were considered not of relevance if the adjusted *p* value was above 0.05.


Metabolite~Age+Sex+Diet+Weight+(1∣ID) (2)

Metabolite~Age+I(Age^2)+Sex+Diet+Weight+(1∣ID) (3)

## 3 Results

Data from 21 cats was included in the analyses, with numbers of samples varying depending on sample availability and analysis (Supplementary Table 1). Bodyweight increased during kitten growth phase, maintaining a healthy growth trajectory. Once reaching adulthood, bodyweight was maintained, showing seasonal variation as previously described (20, 21).

After QC based feature cleaning, the following number of variables was available for each respective data set: plasma metabolomics; 64, plasma lipidomics; 278, fecal metabolomics; 66, fecal lipidomics; 401 and serum biochemistry; 23. Box-cox transformations showed no improvement on data normality for any of the data sets: all transformations resulted in fewer variables passing the normality criterion of the Shapiro–Wilk test.

### 3.1 Metabolomics and lipidomics

Age-related relationships are visible in the PCA scores plots for both fecal datasets (Supplementary Figure 1), along the first component of the fecal metabolomics data and second component of the fecal lipidomics data.

Metabolomics and lipidomics data were corrected for intensity drift, though a small analytical batch effect was still present in both metabolomics datasets. Batch and diet are confounded in the fecal lipidomics data, but as the QC samples are well clustered, the observed batch effect is more likely due to diet type. This did not confound age, the primary outcome for this study (Supplementary Figure 2). Due to the numbers of metabolites and lipids detected, the discussion is focused on the 10 most important and statistically significant metabolites based on RM-ASCA+ modeling for each matrix and the same for lipids.

#### 3.1.1 Plasma metabolomics

The first component of the PCA on the fixed effect matrix explained 41.7% of the age-related variance in the plasma metabolome. RM-ASCA+ modeling identified a distinct U-pattern (Figure 1) in the first principal component of plasma metabolomics data, with loadings largely being driven by organic and amino acids lactic acid and aspartic acid, which showed high concentrations at early and late life stages and decreased during the mid-life stage (2–7 years old). Further metabolites with high positive loadings include purine derivatives xanthine and hypoxanthine, uracil, 2-hydroxyisobutyric acid and phosphoric acid. Inversely, concentrations of glucose and fructose were higher during mid-life.

**Figure 1:**
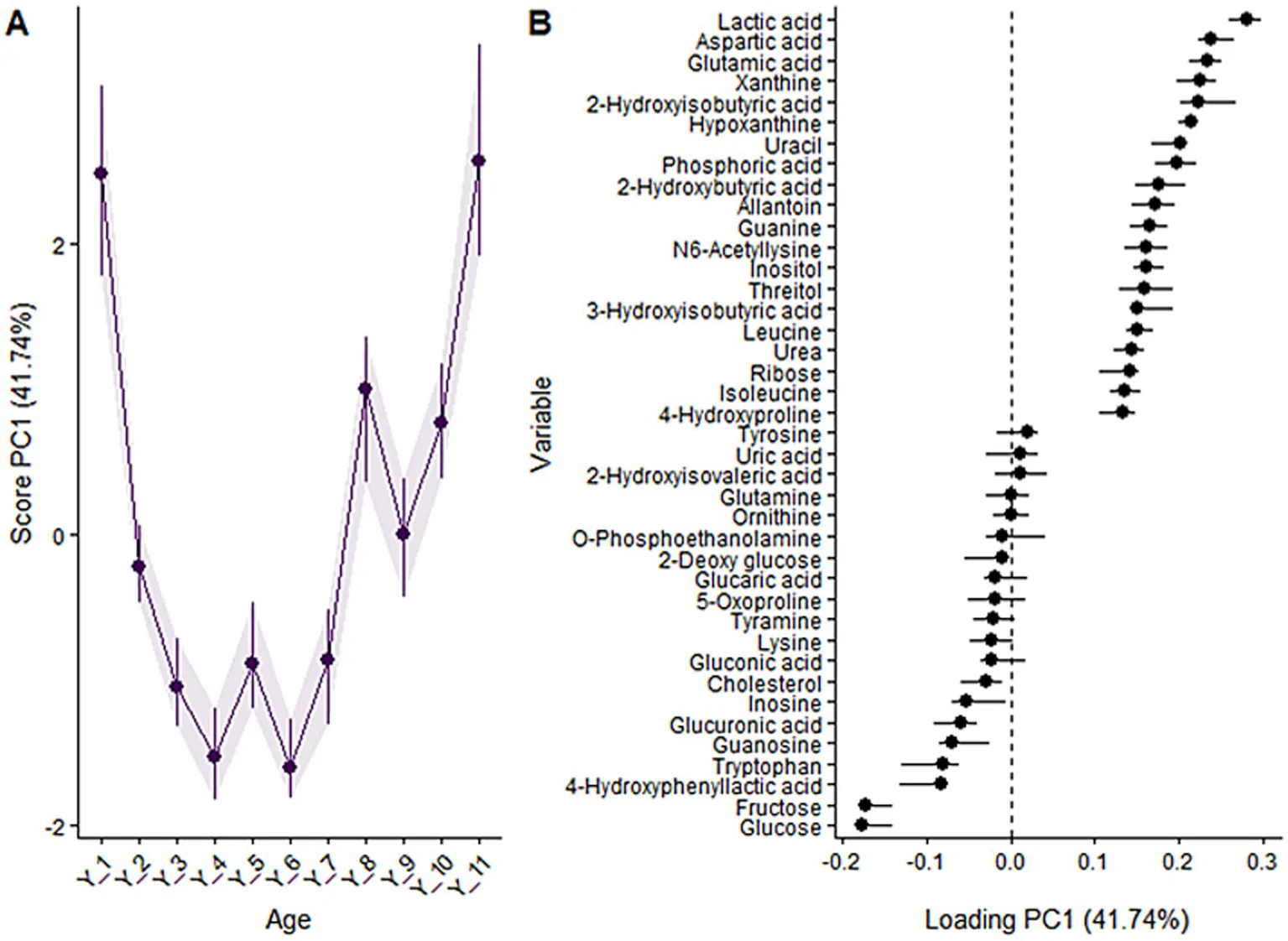
ALASCA output for plasma metabolomics. **(A)** PC1 score trajectory of the fixed effects over time. Error bars produced from bootstrapping (*n* = 1,000). **(B)** Loadings for the top 20 most negative and positive scoring metabolites on PC1. To interpret these plots, plot **(A)** is a representation of the largest sources of variation in the age-related fixed effects from all variables in the dataset while plot **(B)** shows the top individual contributions to the scores showed in plot **(A)**. Positive values in plot **(B)** follow the “U” shaped trend in plot **(A)**, while negative values follow an inverse “U” shape.

Fixed effects were extracted for the metabolites with the highest RM-ASCA+ loadings (absolute value) and show the expected change of the feature intensity as compared to the baseline (year 1) after accounting for the covariates. The fixed effects patterns indicate that the metabolites follow a similar pattern to the score of the first principal component, with minor individual deviations (Supplementary Figure 3). In the case of glucose and fructose, with a large negative loading, the inverse pattern is seen. The fixed effects in all models showed a quadratic pattern and thus linear mixed models were evaluated with a quadratic term as described in Equation 3 to confirm the relationship with age.

Linear mixed modeling found that the majority (9 out of 10) of the metabolites with highest loadings on the first principal component were significant after outlier removal and multiple testing correction with the exception of glutamic acid (Table 1). Most metabolites showed similar or lower *p* values in the quadratic term compared to the linear term, reflecting the non-linear U-shape observed in the RM-ASCA+ modeling.

**Table 1:** Linear mixed model output for plasma metabolomics.

Metabolite	Estimate		*p* value		Adjusted *p* value	
	Age	Age^2^	Age	Age^2^	Age	Age^2^
Lactic acid	−82,425	8,254	0.0027	<0.001	0.019	0.0025
Aspartic acid	−994.3	100.1	0.016	0.0022	0.064	0.017
Glutamic acid	−928.1	77.33	0.176	0.15	0.56	0.5
Xanthine	−24.19	2.687	0.04	0.0041	0.14	0.023
2-Hydroxyisobutyric acid	−1,354	107.8	<0.001	<0.001	<0.001	<0.001
Glucose	191,986	−17,632	0.0066	0.0016	0.035	0.017
Fructose	4,418	−408	0.011	0.0032	0.045	0.02
Hypoxanthine	−1706	157.7	0.0088	0.0022	0.043	0.017
Uracil	−52.93	4.755	0.022	0.0094	0.082	0.043
Phosphoric acid	−49,458	3,584	<0.001	<0.001	0.0015	0.0025

Estimates and *p* values (raw and false discovery rate adjusted) are presented for the 10 metabolites with the largest absolute loadings from plasma metabolomics dataset after removal of outliers based on residuals. FDR *p* values were adjusted with *n* = 64 variables. Age was modeled with linear (“Age”) and quadratic (“Age^2^”) terms based on the metabolite relationship with aging.

#### 3.1.2 Plasma lipidomics

Plasma lipidomics data had an initial plateau from 1 to 7 years, followed by a downwards trajectory from year 8, with the first component explaining 40.9% of the total variance associated with age within the plasma lipidome (Figure 2). This is largely explained by the decline of phosphatidylethanolamines (PE), while14 phosphatidylcholines (PC) decreased with age and 10 increased with age. It is also notable that desmosterol [ST (27:2-O)] has a strong negative loading on the first principal component, suggesting that it increases with age. This is mirrored in the individual fixed effects of the top ranked plasma lipids (Supplementary Figure 4). Desmosterol increased almost quadratically throughout all the 11 years and PC-O (16:1, 18:1) displays a U-like shape having an increase in years 3–5 followed by a decrease after with a rapid drop between years 7 and 8.

**Figure 2:**
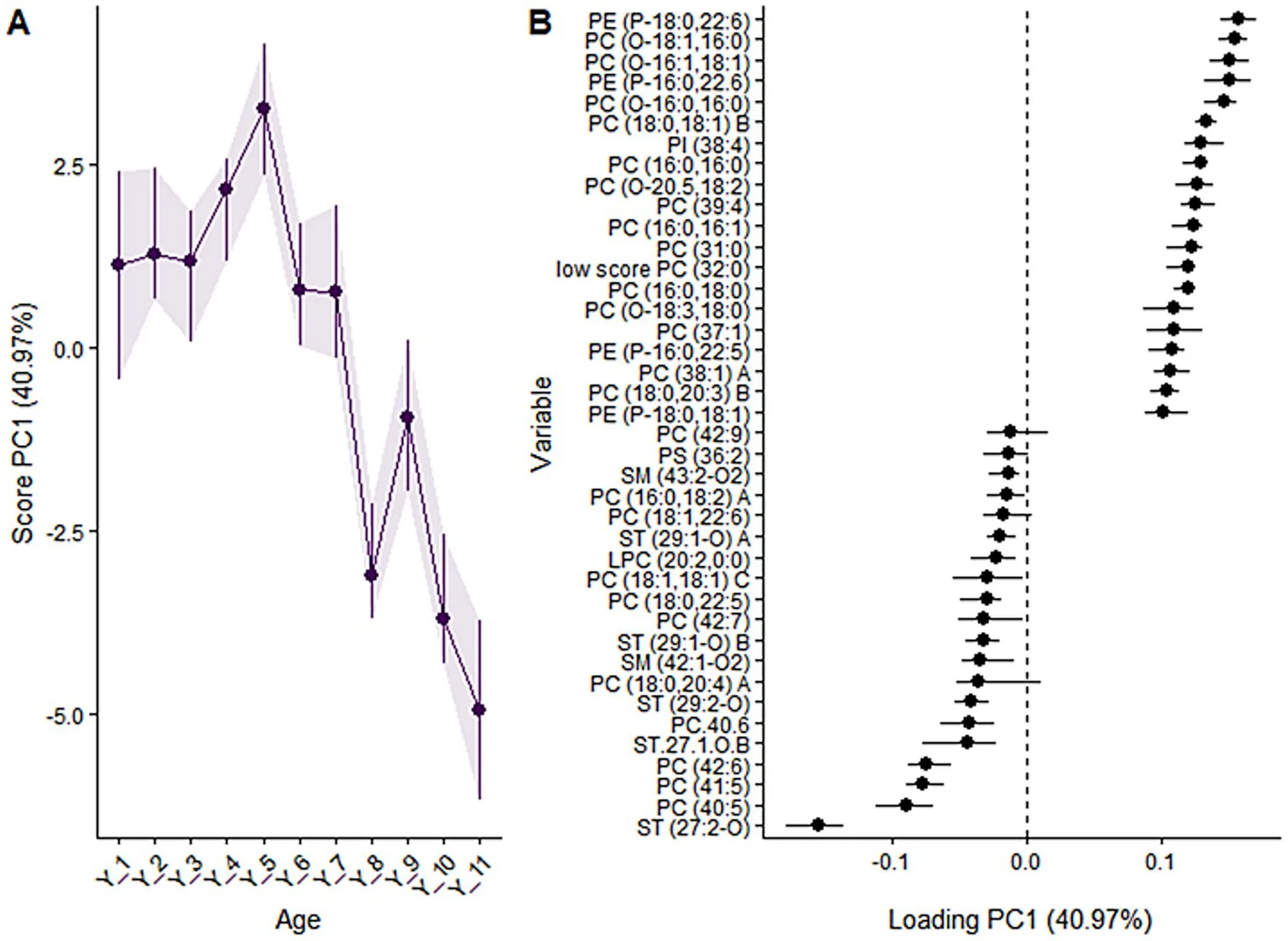
ALASCA output for plasma lipidomics. **(A)** PC1 score trajectory of the fixed effects over time. Error bars produced from bootstrapping (*n* = 1,000). **(B)** Loadings for the top 20 most negative and positive scoring metabolites on PC1. To interpret these plots, plot **(A)** is a representation of the largest sources of variation in the age- related fixed effects from all variables in the dataset while plot **(B)** shows the top individual contributions to the scores showed in plot **(A)**. LPC, Lysophosphatidylcholine; PC, Phosphatidylcholine; PE, Phosphatidylethanolamine; PI, Phosphatidylinositol; PS, Phosphatidylserine; SM, Sphingomyelin; ST, Sterol. ST (27:2-O) has been tentatively identified as desmosterol.

All plasma lipids that were modeled using linear mixed models without the quadratic term were strongly associated with age (*p* < 10^−6^; Table 2). Removal of these outliers did not attenuate these associations. For plasma lipids modeled with the quadratic term [PC-O (16:1, 18:1) and ST (27:2-O)], removal of outliers strongly attenuated the association for the two lipids, with only PC-O (16:1, 18:1) remaining statistically related with age (*p* = 0.012; Table 2).

**Table 2:** Linear mixed model output for plasma lipidomics.

Metabolite	Estimate		*p* value		Adjusted *p* value	
	Age	Age^2^	Age	Age^2^	Age	Age^2^
PE-P (18:0, 22:6)	−4,930	NA	<0.001	NA	<0.001	NA
PC-O (18:1, 16:0)	−14,053	NA	<0.001	NA	<0.001	NA
ST 27:2-O	411.6	1,035	0.95	0.046	1	1
PC-O (16:1, 18:1)	15,444	−2070	0.033	<0.001	0.91	0.012
PE-P (16:0, 22:6)	−4,165	NA	<0.001	NA	<0.001	NA
PC-O (16:0, 16:0)	−2,929	NA	<0.001	NA	<0.001	NA
PC (18:0, 18:1)	−182,398	NA	<0.001	NA	<0.001	NA
PI-38:4	−15,071	NA	<0.001	NA	<0.001	NA
PC (16:0, 16:0)	−22,330	NA	<0.001	NA	<0.001	NA
PC-O (20:5, 18:2)	−2,274	NA	<0.001	NA	<0.001	NA

Estimates and *p* values (raw and false discovery rate adjusted) are presented for the 10 metabolites with the largest absolute loadings from plasma lipidomics dataset after removal of outliers based on residuals. FDR *p* values were adjusted with *n* = 278 variables. Age was modeled with linear (“Age”) and quadratic (“Age^2^”) terms based on the apparent lipid relationship with aging. PE, Phosphatidylethanolamine; PC, Phosphatidylcholine; PI, Phosphatidylinositol; ST, Sterol. NA: model not evaluated for a quadratic relationship.

#### 3.1.3 Fecal metabolomics

The first component from RM-ASCA^+^ explained 51.5% of the variance in the fecal metabolome’s age-related effects. This component had a high score during the first 5 years, followed by a steep drop at year 6 and a low score through years 6 to 11 (Figure 3). This suggests a high concentration of several compounds related to nucleotides (thymine, hypoxanthine, xanthine and uracil) in the first years, dropping lower later in life. Anthranilic acid, 3-hydroxy-anthranilic acid, 4-aminobenzoic acid, nicotinic acid (vitamin B_3_) and several other metabolites relating to amino acid metabolism also had a high loading on the first principal component, thus having similar age-related estimates. The majority of the fixed effects of the individual metabolites showed higher values in the first 5 years and lower values in the subsequent 6 years, albeit with some individual variation [e.g., uracil in year 3, and 4-aminobenzoic acid in year 1 (Supplementary Figure 5)]. 4-hydroxyphenyllactic acid was the only major exception showing higher fixed effects only in years 4 and 5. 4-Hydroxyphenyllactic acid was therefore modeled using a quadratic term in the linear mixed models.

**Figure 3:**
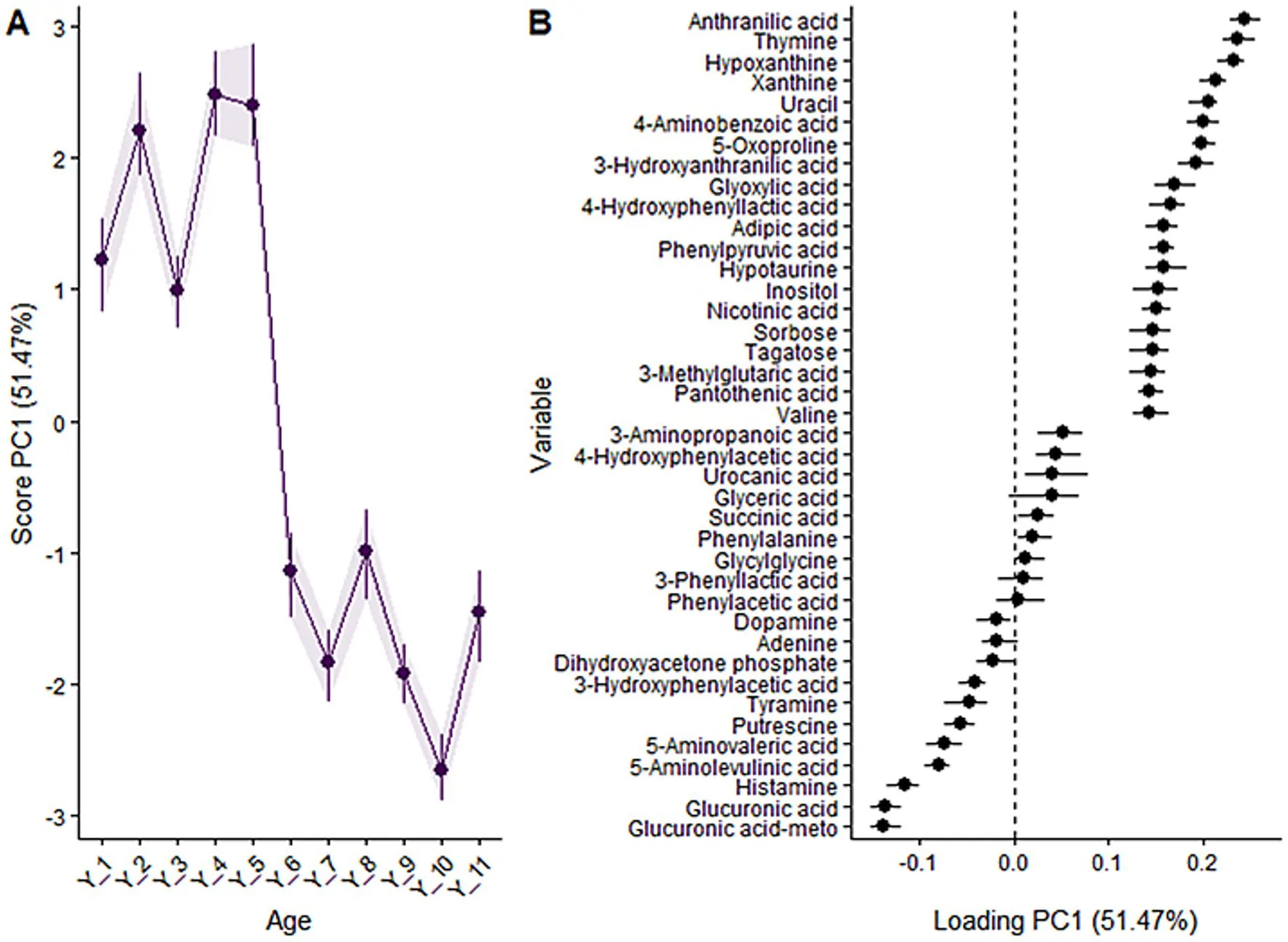
ALASCA output for fecal metabolomics. **(A)** PC1 score trajectory of the fixed effects over time. Error bars produced from bootstrapping (*n* = 1,000). **(B)** Loadings for the top 20 most negative and positive scoring metabolites on PC1. To interpret these plots, plot **(A)** is a representation of the largest sources of variation in the age-related fixed effects from all variables in the dataset while plot **(B)** shows the top individual contributions to the scores showed in plot **(A)**.

All 10 fecal metabolites included in linear mixed modeling differed over time (Table 3). Higher *p*-values could be observed for 4-hydroxyphenyllactic acid, and although the quadratic term is still significant after *p*-value adjustment, care should be taken in the interpretation as there were significant heteroscedasticity issues in the residuals for this metabolite.

**Table 3:** Linear mixed model output for fecal metabolomics.

Metabolite	Estimate		*p* value		Adjusted *p* value	
	Age	Age^2^	Age	Age^2^	Age	Age^2^
Anthranilic acid	−1.411	NA	<0.001	NA	<0.001	NA
Thymine	−26.62	NA	<0.001	NA	<0.001	NA
Hypoxanthine	−78.30	NA	<0.001	NA	<0.001	NA
Xanthine	−101.7	NA	<0.001	NA	<0.001	NA
Uracil	−53.87	NA	<0.001	NA	0.0047	NA
4-Aminobenzoic acid^†^	−1.296	NA	<0.001	NA	<0.001	NA
5-Oxoproline	−591.8	NA	<0.001	NA	<0.001	NA
3-Hydroxyanthranilic acid^†^	−1.512	NA	<0.001	NA	<0.001	NA
Glyoxylic acid	−13.50	NA	<0.001	NA	<0.001	NA
4-Hydroxyphenyllactic acid^†^	2.480	−0.2910	0.050	0.0046	0.30	0.03

Estimates and *p* values (raw and false discovery rate adjusted) are presented for the 10 metabolites with the largest absolute loadings from fecal metabolomics dataset after removal of outliers based on residuals. FDR *p* values were adjusted with *n* = 278 variables. Age was modeled with linear (“Age”) and quadratic (“Age^2^”) terms based on the apparent metabolite relationship with aging. NA: model not evaluated for a quadratic relationship. † Heteroscedasticity issues present after outlier removal.

#### 3.1.4 Fecal lipidomics

The first principal component obtained using data from the fecal lipidomics shows a similar U-shape to that of the plasma metabolomics data while explaining 38% of the total variance in the fecal lipidome due to age, with scores being notably higher in year 1, 10 and 11, and a dip from year 3–5 (Figure 4). Several sphingomyelin entities have a high loading on the first principal component, together with three different triglycerides with two or three C18:3 (likely α-linolenic acid) fatty acids attached. Coenzyme Q8, Q9, Q10 and Q11 together with several bile acids have large negative loadings, suggesting these are present at higher concentrations between years 2–9. Eight of the ten individual fixed effects in the fecal lipidomics samples showed quadratic patterns. The two exceptions were the triglycerides (18:3, 18:3, 18:3) and (18:2, 18:3, 18:3), that both only showed an increase in year 11 (Supplementary Figure 6). These triglycerides were investigated both with and without the quadratic term and only the results from analysis with the quadratic term are reported.

**Figure 4:**
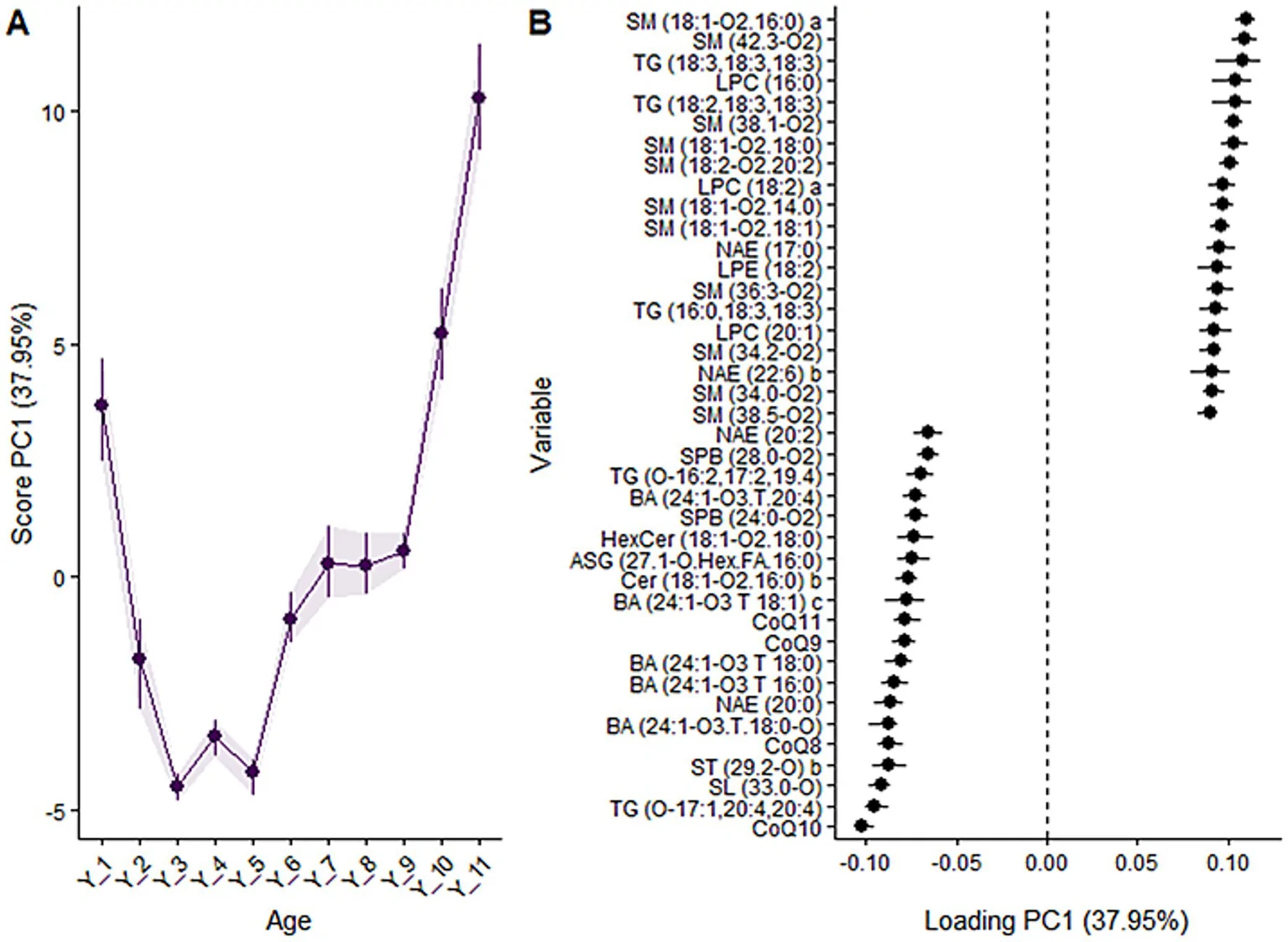
ALASCA output for fecal lipidomics. **(A)** PC1 score trajectory of the fixed effects over time. Error bars produced from bootstrapping (*n* = 1,000). **(B)** Loadings for the top 20 most negative and positive scoring metabolites on PC1. To interpret these plots, plot **(A)** is a representation of the largest sources of variation in the age related fixed effects from all variables in the dataset while plot **(B)** shows the top individual contributions to the scores showed in plot **(A)**. ASG, Acylhexosyl; BA, Bile acid; Cer, Ceramide; CoQ, Coenzyme Q; HexCer, Hexosylceramide; LPC, Lysophosphatidylcholine; LPE, Lysophosphatidylethanolamine; NAE, N-acyl ethanolamine; SL, Sulfonolipid; SM, Sphingomyelin; SPB, Sphingoid base; ST, Sterol; TG, Triglyceride.

Linear mixed models detected significant time dependent changes over time for all of the top metabolites except the two triglycerides (18:3, 18:3, 18:3) and (18:2, 18:3, 18:3) (Table 4). These triglycerides also showed major issues in heteroscedasticity and non-normal residual distributions (data not shown).

**Table 4:** Linear mixed model output for fecal lipidomics.

Metabolite	Estimate		*p* value		Adjusted *p* value	
	Age	Age^2^	Age	Age^2^	Age	Age^2^
SM (18:1-O2, 16:0)	−15,121	1,431	<0.001	<0.001	<0.001	<0.001
SM (42:3-O2)	−364	35.77	<0.001	<0.001	<0.001	<0.001
TG (18:3, 18:3, 18:3)^†^	−17.46	5.65	0.68	0.10	1	1
LPC-O 16:0	−520.2	55.97	<0.001	<0.001	0.0026	<0.001
TG (18:2, 18:3, 18:3)^†^	−23.33	10.54	0.78	0.13	1	1
SM (38:1-O2)	−825.8	76.11	<0.001	<0.001	<0.001	<0.001
SM (18:1-O2, 18:0)	−5,537	523.1	<0.001	<0.001	<0.001	<0.001
SM (18:2-O2, 20:2)	−1,552	135.8	<0.001	<0.001	<0.001	<0.001
TG (O-17:1, 20:4, 20:4)	345.8	−38.17	<0.001	<0.001	<0.001	<0.001
CoQ10	5,561	−655.3	<0.001	<0.001	<0.001	<0.001

Estimates and *p* values (raw and false discovery rate adjusted) are presented for the 10 metabolites with the largest absolute loadings from fecal lipidomics dataset after removal of outliers based on residuals. FDR *p* values were adjusted with *n* = 406 variables. Age was modeled with linear (“Age”) and quadratic (“Age^2^”) terms based on the apparent lipid relationship with aging. SM, Sphingomyelin; LPC, Lysophosphatidylcholine; TG, Triglyceride; CoQ, Coenzyme Q. † Heteroscedasticity and normality issues present after outlier removal.

### 3.2 Serum biochemistry

The biochemical and ELISA analyses showed a strong downwards near-linear trend in the first principal component (Figure 5). The first component explained 62.6% of the total variance among biochemical markers related to age in the fixed effects matrix. Changes were mostly driven by a decrease in albumin:globulin ratio, albumin, calcium and phosphate, and an increase of globulin, aspartate transaminase (AST), alanine aminotransferase (ALT), total protein, urea and bilirubin with age. Among those, only albumin and globulin mean values were outside the reference range – albumin below reference range in years 9–11 and globulin above reference range in year 11. AST and ALT only had some outliers above adult reference range at year 11, while phosphate observed range was marginally above adult reference range at year 1 (Supplementary Datasheet 1). Examining the fixed effects of the 10 variables with highest loadings on the first principal component of the RM-ASCA+ model, most variables show a similar pattern to that of the scores, with the exception of phosphate, that is present at high concentrations in year one but then stays at a constant relatively lower concentration from year 2 onwards (Supplementary Figure 7). All biochemical markers were investigated without the quadratic term apart from phosphate, which was investigated using both versions. Although alkaline phosphatase did not have a significant effect of age in univariate analysis, it showed a steep drop at year 2 after being above the adult reference range in year 1, while showing a slight increase after year 8 (Supplementary Datasheet 1). This was supported by a strong loading in the second principal component, with the same drop between years 1 and 2 (data not shown).

**Figure 5:**
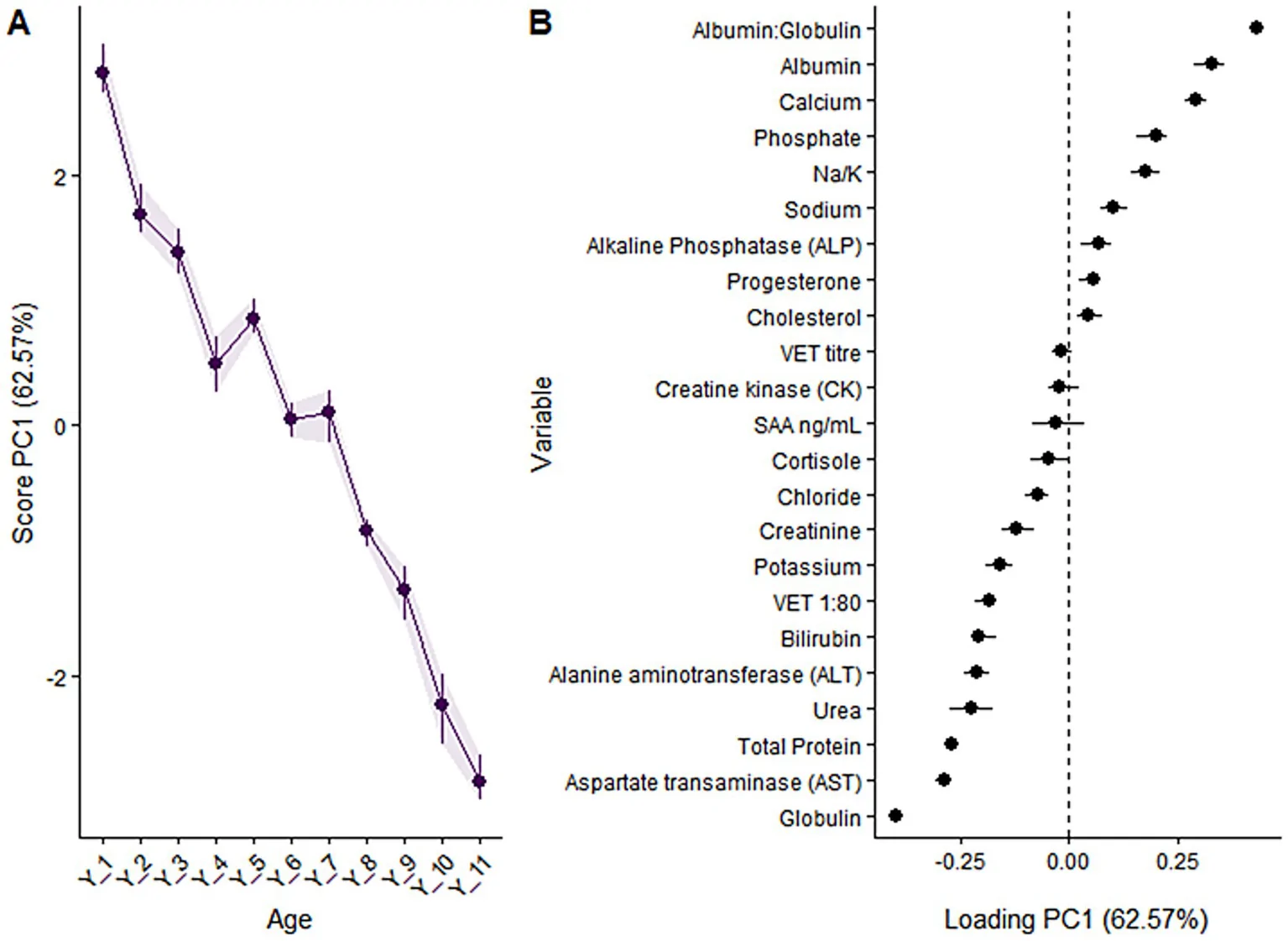
ALASCA output for serum biochemistry. **(A)** PC1 score trajectory of the fixed effects over time. Error bars produced from bootstrapping (*n* = 1,000). **(B)** Loadings for the top 20 most negative and positive scoring metabolites on PC1. Age was modeled with linear (“Age”) and quadratic (“Age^2^”) terms based on the apparent biochemical marker relationship with aging. SAA, Serum Amyloid A; VET titre and VET 1:80: *Bordetella bronchoseptica* antibodies.

The 10 biochemical markers with the strongest loadings showed nominally significant changes over time in the linear mixed models (Table 5). Outlier removal did not result in any major changes in the estimates.

**Table 5:** Linear mixed model output for biochemical markers.

Metabolite	Estimate	*p* value	Adjusted *p* value
	Age	Age	Age
Albumin:Globulin	−0.0379	<0.001	<0.001
Albumin	−0.6478	<0.001	<0.001
Globulin	1.428	<0.001	<0.001
Aspartate transaminase (AST)	1.101	<0.001	<0.001
Total protein	0.9224	<0.001	<0.001
Calcium	−0.01723	<0.001	<0.001
Phosphate	−0.02037	<0.001	<0.001
Urea	0.1595	<0.001	<0.001
Alanine aminotransferase (ALT)	1.904	<0.001	<0.001
Bilirubin^†^	0.01965	0.0014	0.0032

Estimates and *p* values (raw and false discovery rate adjusted) are presented for the 10 biochemical markers with the largest absolute loadings from biochemical markers dataset after removal of outliers based on residuals. FDR *p* values were adjusted with *n* = 23 variables. Age was modeled with linear (“Age”) terms. No quadratic trends with aging for these markers; quadratic terms not calculated.

### 3.3 Combined plasma and fecal data

To determine which metabolites and lipids from the overall dataset had the strongest relationship with aging, the four metabolomics datasets and serum biochemistry were modeled together. Fecal lipidome features are the most predominant in the first principal component, which included several biochemical markers as well as fecal metabolomics data (Figure 6). In total, 25, 8, 5, and 2 features come from the fecal lipidome, fecal metabolomics, serum biochemistry and plasma metabolomics data respectively, while no features from the plasma lipidome are present. Scores from the first principal component show a high score in years 1 through 5, steeply decreasing at year 6 and continuing to decrease until year 11. Most of the top-ranked features in the combined data are also present in the previous models, but notable additional differences include that fecal N-acylethanolamines appear to have a greater impact on this model than in the fecal lipidome alone, and plasma metabolites kynurenine and isoleucine also increase with age. Furthermore, several triglycerides with one polyunsaturated fatty acid (20:4 or 20:5) in the fecal metabolome increase with age. Univariate analysis additionally identified fecal histamine as increasing with age (Table 6). These metabolites and lipids likely did not follow the predominant pattern for the analyses of plasma or feces alone but were more closely aligned with the overall trajectory of all data combined.

**Figure 6:**
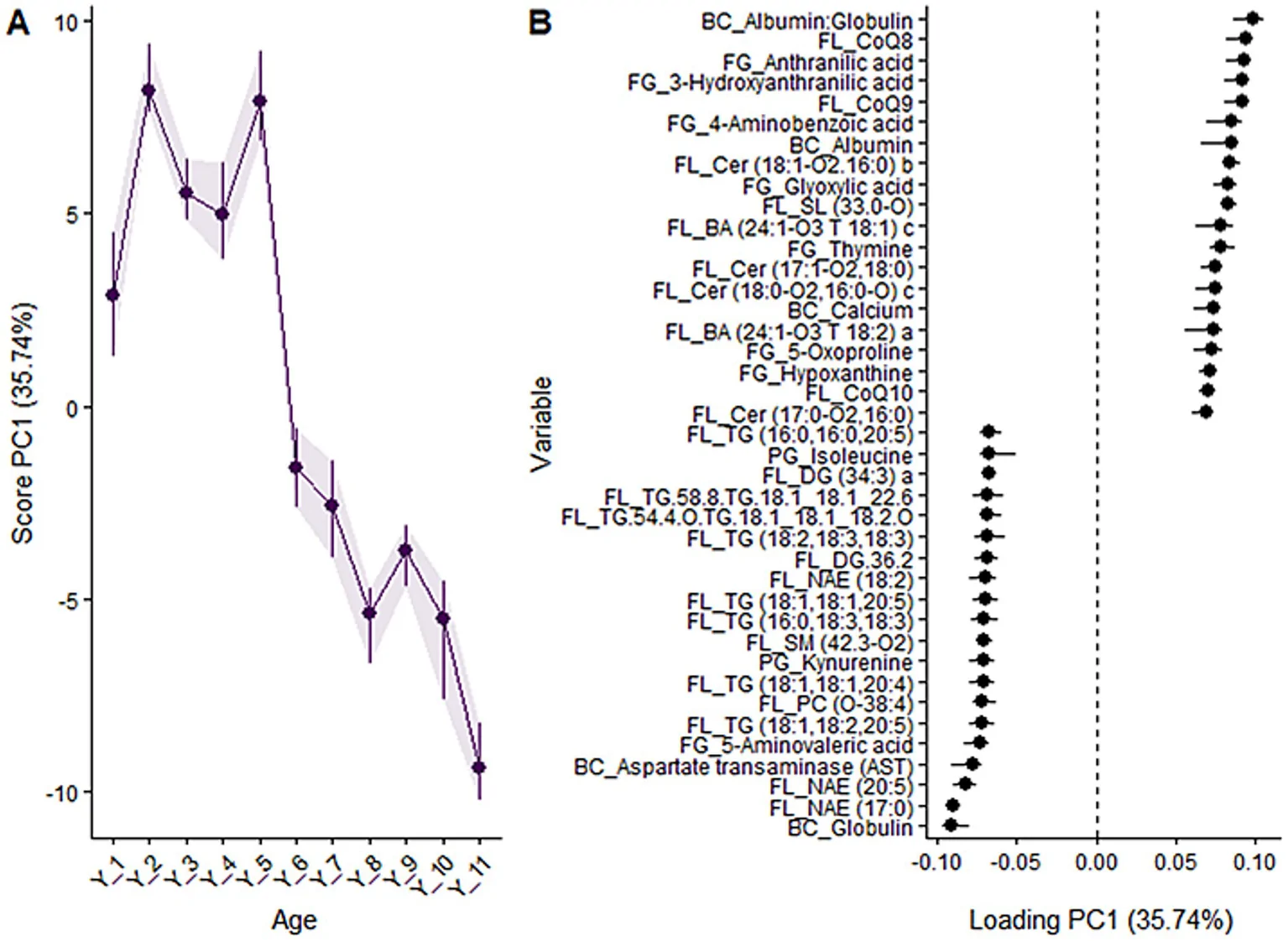
ALASCA output for combined data. **(A)** PC1 score trajectory of the fixed effects over time. Error bars produced from bootstrapping (*n* = 1,000). **(B)** Loadings for the top 20 most negative and positive scoring metabolites on PC1. BC, Biochemical markers; FL, Fecal Lipidome; PL, Plasma Lipidome; FG, Fecal metabolomics; PG, Plasma metabolomics; Cer, Ceramide; SL, Saccharolipid; BA, Bile Acid; TG, Triglyceride; DG, Diglyceride; NAE, N-Acylethanolamine; SM, Sphingomyelin; PC, Phosphatidylcholine.

**Table 6:** Linear mixed model output for combined data.

Metabolite	Estimate	*p* value	Adjusted *p* value
	Age	Age	Age
FM Histamine	46.69	<0.001	0.006
FM 5-Aminovaleric acid	849.5	<0.001	0.009
PM Kynurenine	40.67	<0.001	<0.001
PM Urea	115,971	<0.001	0.003
PM Isoleucine	2,532	<0.001	0.028
FL Tauro-chenodeoxycholate; 18:0	−51.09	<0.001	0.008
FL Tauro-chenodeoxycholate; 18:1	−368.4	<0.001	0.001
FL Tauro-chenodeoxycholate; 16:0	−59.38	<0.001	0.011
FL n-acylethanolamine; 20:5	145.9	<0.001	<0.001
FL n-acylethanolamine; 17:0	280.9	<0.001	<0.001

Estimates and *p* values (raw and false discovery rate adjusted) are presented for the 10 parameters with the largest absolute loadings from the combined data set, after removal of outliers based on residuals. FDR *p* values were adjusted with *n* = 548 variables. No quadratic trends for these compounds; quadratic terms not calculated. FM, fecal metabolomics; PM, plasma metabolomics; FL, fecal lipidomics.

## 4 Discussion

This study is the first to probe metabolic changes in cat aging through a longitudinal design across 11 years, using two sample matrices, and using both metabolomics and lipidomics. Together with serum biochemistry, this provides one of the most holistic overviews of cat metabolic aging to date, covering young adult (1–6 years), mature adult (7–10 years), and the first year of senior (>10 years) life stages (29).

### 4.1 General patterns observed in the datasets

Two main metabolome and lipidome patterns emerged from the data; the first pattern exhibiting roughly linear increases or decreases with age (plasma lipidomics, fecal metabolomics, serum biochemistry and combined data), and the second one a regular-U or inverse-U-shaped pattern, with most changes occurring during the transition in and out of the middle stages of the lifespan (plasma metabolomics and fecal lipidomics). The first pattern suggests progressive or cumulative changes with age that may relate to accumulation of damage or gradual loss of organ function. The second suggests a mid-age range between growth and development that could be interpreted as a “steady state” period of life, while the beginning of the U-shape may relate to maturation into adulthood and the end of the U-shape trajectory may reflect aging alterations occurring more abruptly. The trend for a linear decrease or increase in metabolites, lipids and biochemical markers was strongest in the combined dataset from age 6, although this trend was preceded by a plateau from year 1–5, suggesting a change in overall cat and cat microbiome metabolic state around 6 years.

### 4.2 Changes in plasma metabolome

Plasma metabolome followed the “U”-shaped trend, with a steady period between 3 and 7 years of age. Metabolites most strongly associated with age were lactic acid, glucose and fructose, with higher concentrations of lactic acid alongside lower concentrations of glucose and fructose at early and later life, which may suggest changes to energy metabolism. Fasting duration was not strictly controlled in our study and likely varied from 12 to 18 h, which may be a confounding factor in these results. Our findings in the mature-senior life stages are consistent with previous findings in cats, which were suggested to reflect a shift towards a higher proportion of anaerobic glycolysis and possibly mitochondrial dysfunction (15, 30). Conversely, the findings at growth stages may reflect mitochondrial immaturity. In mice, the content of mitochondrial complex I, a key component of the respiratory chain, increases in tissues during growth, before reaching a plateau at 25 days (31).

Nucleic acid-related compounds xanthine, hypoxanthine, guanine, phosphoric acid, allantoin, and uracil also followed a U-shaped trend in plasma with higher concentrations at younger and older ages, while xanthine, hypoxanthine and uracil had a decreasing linear trend with age in feces. These are key metabolites in the purine and pyrimidine pathways, which are essential for the synthesis, salvage, and catabolism of nucleic acids including DNA and RNA bases. Xanthine and hypoxanthine are then oxidized into uric acid and finally into allantoin. The trend observed could reflect the overall higher turnover of tissues during growth, and a shift towards increased catabolism during the aging process. Other factors such as reduced renal clearance with age or changes in energy requirements and turnover of ATP could also lead to the same metabolites (32). Higher allantoin levels in the older cats could also reflect increased oxidative stress, as an association between allantoin, oxidative stress and aging has been shown in humans (33). Fecal pyrimidines and purines decreased with age, and the difference in pattern suggests that changes to nucleic acid building blocks with age between plasma and feces are not directly related.

If anabolism and catabolism were key drivers of the aging cat metabolome, we would expect to observe the concentration of amino acids in the plasma pool being altered by age (15). In the present study, two non-essential acidic amino acids; aspartic and glutamic acids, were strongly associated with the U-shaped trend with age, and the essential branched chain amino acid isoleucine followed a similar pattern. Aspartic and glutamic acids following a similar trend to lactic acid supports a shift from predominantly aerobic metabolism towards more anaerobic metabolism with age; both are precursors to oxaloacetate, a Krebs cycle intermediate, and less consumed with a lower proportion of aerobic metabolism.

Organic acids 2-hydroxybutryic acid (2HBA), 2-hydroxyisobutyric acid (2HIBA) and 3-hydroxyisobutyric acid (3HIBA) also followed the U-shaped trend in plasma. These three C4 organic acids are metabolic intermediates involved in amino acid turnover and energy metabolism, though with different precursors.3HIBA is an intermediate in valine catabolism (34), while 2HBA is derived from α-ketobutyrate which is a product from methionine trans-sulphuration and threonine catabolism (35–38). Production of 2HBA, and likely 2HIBA are associated with elevated NADH, suggesting that mitochondrial oxidation is saturated or impaired (39). Campbell et al. found that 2HIBA and 3HIBA were increased in the muscle of young and old mice after high intensity exercise relative to low intensity exercise, which was linked biochemically to mitochondrial fat oxidation and remained activated even while other pathways were no longer stimulated during aging (40). In the context of higher concentrations at younger and older ages it may mean more time spent in both amino acid and energy imbalance – i.e. more amino acid building blocks and energy required for growth and maintenance than is available from sources other than amino acids, leading to a greater amount of both amino acid and mitochondrial fat oxidation (35, 40, 41). Underlying reasons for this in aging cats may be overall reduced energy intake (42) or development of sarcopenia (43, 44).

### 4.3 Changes in plasma lipidome

After a relatively stable equilibrium until 7 years of age, plasma lipidome mostly followed a linear decline or increase. Of the 34 lipids that decreased or increased with age, 23 were phosphatidylcholines (PCs). Of the eight PCs that increased with age, five had longer fatty acid substitutions (total 40–42 carbons) and more double bonds (5–6 double bonds), while all the PCs that decreased with age had shorter fatty acid substitutions (total 30–38 carbons) and fewer double bonds (0–2). Exceptions from this were the PC O-38:7 that shared the decreasing trend in age. This difference may reflect changes to both fatty acid synthesis and β-oxidation with age, or a difference in the uptake of dietary fatty acids, given that most dietary fatty acids have chain lengths <20. β-oxidation is the metabolic process that converts fatty acids into energy in the mitochondria. To our knowledge there has been no direct assessment of the effect of age on fat metabolism in cats, though cats do accumulate fat in the liver with age, which can be both a cause and effect of decreased capacity for β-oxidation (45). Our findings may suggest a diminished ability to convert the mobilized lipid pool in plasma to energy is diminished with age, possibly leading to the observed difference in fatty acid chain length in circulating phospholipids. This could have a knock-on effect on membrane phospholipid composition and fluidity. In addition, phospholipids with long-chain polyunsaturated fatty acids have been associated with destabilization of lipid membranes in cell models and rodents, leading to inflammation (46), and fitting with longer-chain PCs increasing with age. Mobilization of phospholipids with arachidonic acid is part of the inflammatory response pathway for synthesizing prostaglandins (47), which are key mediators of inflammatory response (48), and may also explain the observation that circulation of phospholipids changes with age. Intriguingly, circulating phosphatidylcholine has been found to decline with age in both *Caenorhabditis elegans* and humans, with the decline linked to increased mitochondrial dysfunction via changes to mitochondrial network integrity (49). The change to phosphatidylcholine composition with age in our study could support these recent findings on age-related changes to mitochondria; direct measures of hepatic or mitochondrial fatty acid oxidation are warranted to confirm this hypothesis.

### 4.4 Changes in fecal metabolome

Fecal metabolites had a similar trajectory of a decrease/increase over time, though in contrast to plasma lipids, the trajectory suggests two relatively stable plateaus, with the breakpoint occurring between 5 and 6 years of age. Of the metabolites that decreased with age, fecal anthranilic acid and 3-hydroxyanthranilic acid (3-HAA) indicate shifts in tryptophan catabolism in the gut. Both compounds can be produced via gut microbiome or endogenous metabolism of tryptophan via the kynurenine pathway (50). Gut tryptophan metabolites are suggested to play a role as immune modulators in aging and chronic inflammation (51, 52). 3-HAA promotes lifespan through activation of Nrf2/SKN-1, an oxidative stress response transcription factor in rodents and worms (53), and is linked with extended lifespan in several non-feline studies (54). L-Kynurenine, the precursor to 3-HAA, was however found to have the opposite concentration in plasma, with lower concentrations in the early life based on the combined model. This is consistent with a study in humans that found increased tryptophan breakdown products in serum as individuals aged (55), likely linked to association of increased blood kynurenine in humans with age (56). Dysmetabolism of the tryptophan-kynurenine pathway has been linked with disruptions in mitochondrial respiration and energy production (57), supporting other markers in this study relating to possible changes in β-oxidation. Tryptophan metabolism has several branch points, including conversion via kynurenine and quinolinic acid to nicotinic acid, another metabolite found to decrease with age in fecal samples, similar to anthranilic acid and 3-HAA. Nicotinic acid, a vitamer of vitamin B_3_, is not synthesized by cats in sufficient quantities to meet needs due to a high rate of metabolic conversion of tryptophan to glutamate and acetyl-CoA (58, 59). Nicotinic acid is a precursor of the coenzymes NAD^+^ and NADP^+^, which facilitate the activity of a wide range of enzymatic processes including fat, carbohydrate and protein catabolism, cell signaling and DNA repair. Fecal concentration changes of tryptophan metabolites would be expected to be linked to an interaction between diet, nutritional requirements and gut microbiome. Overall, the data points to a life-stage dependent modulation of tryptophan related pathways. Targeted studies focusing on these pathways would be of interest for future studies on feline aging. The association of these tryptophan metabolites suggests that changes to the kynurenine pathway for metabolism of tryptophan is part of the aging process in cats, as for other species, and that this may represent an area of interaction between the gut microbiome and feline host. We note that much research on tryptophan metabolism, gut microbiome and aging focuses on its metabolism to indoles (51), which were not found to differ in this cohort. Whether this is specific to this study or if gut tryptophan metabolism may differ in carnivores should be followed up in future work.

The decrease of the fecal pyrimidines thymine and uracil and the fecal purine derivatives xanthine and hypoxanthine with age suggests a decreased pool of bases for nucleic acid synthesis or alternatively a lower shedding of nucleic acids from the intestinal epithelium or microbiota in older cats. Cell division rate declines with age in the colon of humans, which would support the hypothesis of a lower shedding from the intestinal epithelium, although this is not the case for mice (60).

Several other pathways appear to have alterations reflected in the fecal metabolome. Among those, histamine was elevated when the cats were older. Histamine-secreting bacteria are present within the human gut microbiota, and their levels are increased in some immune-mediated diseases (61). Histamine may be seen as a signaling pathway linking microbial alterations to chronic inflammation in these disorders, both by direct action on histamine receptors H1R, H2R, and H4R and by interaction with other microbial metabolites – such as short-chain fatty acids (SCFAs) and tryptophan derivatives – which forms an intricate metabolic–inflammatory network (61, 62).

Other fecal metabolites associated with amino acid metabolism that increased with age included 5-aminovaleric acid, a gut microbial catabolism product of lysine metabolism. A possible explanation for the observed increase in amino acid catabolites in feces from older cats could be the apparent decreased protein digestibility with age (21), which could make more protein (and amino acids) available for catabolism by the gut microbiome. In contrast, fecal concentrations of phenylpyruvic acid, a microbial breakdown product of phenylalanine (63), 5-oxoproline, a metabolite of glutamine metabolism and linked to glutathione synthesis (64), and glyoxylic acid, a breakdown product of hydroxyproline (65), decreased with age. These divergent patterns of amino acid breakdown products suggest that the relationship with aging is more complex than can be explained by increased availability of protein for catabolism by the gut microbiome. Compounds measured in the fecal metabolome can come from dietary sources, gut microbiota, shed epithelial cells, and intestinal secretions. A better understanding of these sources of variability, for example through analysis of microbial composition and function could help to better understand the observed changes.

### 4.5 Changes in fecal lipidome

The fecal lipidome followed a U-shaped trend with age, with two possible plateaus at 3 to 5 years and 6 to 9 years, followed by a sharp rise after 9 years. Of the lipids with the highest fecal excretion at young and old ages were sphingomyelins (SM) and lysophospholipids. The implications of SMs in feces are not well studied, but research in humans suggests the most likely source is incomplete digestion of dietary SM as well as intestinal cell turnover (66, 67). It has been hypothesized that increased fecal concentrations of SMs could reflect underlying inflammatory and hepatobiliary processes in human infants (68), but little is known about their role in companion animals. In cats, elevated SMs were detected in higher concentrations in cats with chronic enteropathy, supporting a link with intestinal inflammation (69). Lysophospholipids are phospholipids with only one fatty acid chain, and may be associated with diet, though, like sphingomyelins, have been associated with intestinal inflammation in cats with chronic enteropathy (69).

The fecal lipids that had an inverse-U-shaped relationship with aging comprised largely of Coenzyme Q derivatives and bile acids. Coenzyme Q10 (CoQ10) is a common component of cellular membranes and plays many roles in biology, including in mitochondrial ATP production, part of the endogenous antioxidative system, and may play a role in cellular redox signaling (70). CoQ10 is also cofactor for mitochondrial dehydrogenases involved in fatty acid metabolism (71). Mammalian data suggests that there is a decrease in tissue CoQ10 with age (72), though this relationship has not been reported in feces. Fecal CoQ10 has been linked to increases in short chain fatty acid concentrations in rodents (73). Coenzymes Q8, 9 and 11 differ from CoQ10 in the number of isoprenoid units, and are reported in both bacteria and food, especially vegetable oils and meat (74). Relevant for aging, CoQ10 has previously been reported to increase cell signaling associated with inflammation in humans and rodents (72) and to increase antioxidant enzyme levels and to reduce oxidative stress markers in humans with coronary artery disease (75).

Bile acids, synthesized in the liver from cholesterol, are stored in the gallbladder and released during digestion to solubilize fats in the intestinal lumen so they can be absorbed across the intestine. Most bile acids are recycled via enterohepatic circulation, though a portion are excreted in the feces. In cats, the primary bile acids cholic and chenodeoxycholic acid are mainly conjugated with taurine (76). In this study five fecal bile acids carrying fatty acids 16:0, 18:0, ether-linked 18:0, 18:1, and 20:4, likely derived from the conjugated primary bile acid taurochenodeoxycholic acid, were associated with aging. The decrease of these fecal bile acids in the second half of the inverse U-shape may reflect an overall alteration of the ability to digest fat which has been suggested to occur with age, possibly in link with chronic inflammation (30, 77). It could also reflect decreased β-oxidation, in keeping with our hypothesis for higher concentrations of longer-chain phospholipids in plasma with aging. Notably, no secondary bile acids (metabolized by gut microbiota) were associated with aging in this study.

Fecal N-acylethanolamines (NAEs), signaling lipids involved in inflammatory processes, endocannabinoid signaling and appetite regulation (78), had a large impact on the combined analysis and increased with age. This is likely because their concentration profiles aligned more closely to the variance patterns associated with the integrated dataset than the pattern of the fecal lipidomic data set alone. Fecal NAEs have been shown to be more closely related to diet (79) and the gut microbiome composition, than plasma concentrations of the same compounds (80). During the sampling period, cats consumed the same format, and commercial brand of petfood, age-related changes in NAE are likely to be caused to by changes in the gut environment, though given their role in stimulating anti-inflammatory response (78), this may also reflect a wider response to inflammaging.

### 4.6 Changes in serum biochemistry

The serum biochemical markers most strongly associated with age in this cohort were albumin and globulin, with a decrease with age of the albumin:globulin ratio paralleled by a decrease in albumin and increase in globulins. Albumin is synthesized by the liver and has a vital role in maintaining osmotic balance. Globulins encompass different groups of proteins: the liver produces several α- and β-globulins, including many acute-phase proteins, while γ-globulins (immunoglobulins) are produced by lymphocytes and plasmacytes. During inflammation, the production of both acute-phase proteins and immunoglobulins increase, and serum albumin decreases through cytokine mediated suppression of synthesis and increased breakdown (81). While Pye and colleagues (13) found that serum albumin was increased in mature and senior life stages of cats (15), our findings are consistent with two studies that observed lower serum albumin in older cats (82, 83), underpinning the hypothesis of inflammaging being an important process in aging cats (8, 15).

Other serum biochemical markers were largely within normal ranges throughout the 11-year duration of the study. Biochemical markers are usually used to identify disease, and our observation supports that the cats were clinically healthy. Alanine aminotransferase and aspartate transaminase increased with age, with variation notably larger by 11 years of age. This may reflect subclinical structural alteration of liver, muscle and/or cardiac tissue, possibly linked with the inflammaging hypothesis. Plasma alkaline phosphatase was above the adult reference range in year 1 and decreased steeply by year 2, while showing a slight increase after year 8. The high levels of alkaline phosphatase at year 1 likely reflect bone growth, consistent with the relatively high levels of serum calcium and phosphate at the same period. A higher reference range for alkaline phosphatase was previously observed in kittens between birth and 8 weeks of age, as compared with adult reference range (84). Plasma urea increasing towards the end of the study period suggests a decrease in kidney function. A higher plasma urea concentration in senior compared to mature cats was also observed in a cross-sectional (83) and a longitudinal study (15).

### 4.7 Temporality of changes

Plasma metabolomics and fecal lipidomics trajectories were suggestive of a metabolic “breakpoint” at 2 years of age, which may indicate that the maturation of metabolic processes is reached later than organ maturity (85). A second breakpoint was observed in all datasets at six-eight years. A retrospective analysis of dual-energy x-ray absorptiometry data from over 1,000 cats aged from below 1 year to 16.9 years showed that lean mass increases until a peak at age 4.5 after which it starts to decrease, while fat mass peaks at age 7.5 and then decreases (86). Both lean mass and fat mass are metabolically active and may differently affect plasma and fecal metabolome/lipidome. In our results however, fecal metabolome and lipidome shifts occurred earlier than in plasma, and we speculate this could reflect that age-related gut microbiota changes happen earlier than host metabolism. The present study does not include gut microbiota analysis and we cannot presume a causal relationship between these respective findings, though the concept of a gut microbiota–organ axis in relation to aging is well established in human gerontological research (87–89). In line with this concept of gut microbiota–organ axis, we would have expected to see a breakpoint in plasma biochemistry around this period as well; it was not the case as with few exceptions, most biomarkers followed a linear trend over our observed age range. We have not observed any further metabolic breakpoint in our study however the upper end of our observed age range was 11 years, at the cusp of likely late-age related changes.

### 4.8 Biological themes drawn from our findings

Three main hallmarks of aging have emerged from our data, which are known to be intricately interconnected, rather than having a simple causal relationship. The first one is inflammaging, supported by the following linear changes: a decrease in plasma albumin, an increase in plasma globulins, and an increase in fecal inflammatory mediators like histamine and N-acylethanolamines. The second is mitochondrial dysfunction and a shift in energy metabolism, supported by increased chain length of plasma phosphatidylcholines, increased plasma levels of hydroxy-butyric acids and of lactic acid, decreased plasma levels of glucose, as well as decreased levels of fecal coenzyme Q, at the end of the study span. Most of these changes (with the exception of phosphatidylcholines) followed the U-shape, meaning that levels of these metabolites were comparable between growth and mature-senior stages, as opposed to the adult stage, which may reflect mitochondrial immaturity during growth.

Thirdly, the data tells a divergent story between the host and the gut environment, suggesting that the aging gut microbiome follows a distinct metabolic trajectory, with different metabolites observed in plasma and feces, or opposite trends of the same metabolites. The most striking element here was the tryptophan pathway, with a linear increase of plasma kynurenine and a linear decrease of fecal anthranilic acid and 3-hydroxyanthranilic acid across observed life span. Tryptophan metabolism has been implicated in all three themes above (inflammaging, mitochondrial dysfunction and aging gut microbiota) and in various age-related diseases (90, 91).

An additional finding was the U-shape trajectory of several nucleobases and their derivatives, as well as some amino-acids and urea, in the plasma metabolomic dataset, which we hypothesize to reflect a high tissue turnover during growth, and a tendency to tissue wastage near mature-senior life stage.

### 4.9 Study limitations and future work

The main limitation of this study is the relatively small number of cats, while its main strength is that the duration of longitudinal follow-up is longer than previous reports on aging. Our follow-up ended at 11 years of age, just at the start of senior life stage, so that we do not know whether the trends observed from the age of 6 to 8 years would continue, accelerate or plateau later in life. A longer follow-up would also have allowed us to differentiate effects of physiological versus pathological aging; in our study all cats remained apparently healthy over the studied period and were still free of apparent age-related disease at the time of data analysis. Additional measurements of function such as cognitive assessment or evaluation of physical function could have provided interesting insights in metabolomic changes associated with pre-frailty. Activity and food intake were also not measured in this study, both factors which may impact metabolome with aging. Other studies have suggested that while kittens and junior cats had higher physical activity, prime, mature and senior cats had similar rates (92). Another factor which could not be controlled and was therefore not included in the statistical analysis is the exact duration of fasting (ranging from an estimated 12 to 18 h depending on each cat). Fasting duration can substantially influence circulating metabolites and lipid species and may thus be a confounding factor in our study. Additionally, this study was conducted in a population of research cats, therefore the results may be limited in their generalizability to a wider domestic cat population.

With a complex dataset that is both multivariate and time-based, compromises for the data analysis are necessary. We have used RM-ASCA^+^ modeling, treating age as a categorical factor rather than a continuous variable, which allows the method to capture complex, non-linear patterns of change across the measured lifespan that would not necessarily be detected using linear or exponential regression approaches. While this increases flexibility for exploratory analysis, it comes at the cost of reduced interpretability of directional trends and diminished statistical power for detecting gradual linear or quadratic changes that may be of interest in a hypothesis-driven study. Most of the strongest associations had strong linear or quadratic associations with age and could be modeled as such. Incorporation of fixed effects of known confounders and individual cats as random intercepts allowed the age effect to be isolated and visualized without contributions from unwanted sources of variance. From this perspective, RM-ASCA^+^ is a suitable modeling method and has enabled clear patterns to be deduced. Not all trends detected using RM-ASCA^+^ held with univariate modeling, underlining the importance of using more than one statistical method to assess changes, especially in multivariate datasets. Pathway analysis has not been included in this work due to the limitations and pitfalls of applying tools developed for high coverage genomic workflows to low coverage metabolomics analyses (93).

Future work on understanding what changes occur in cats with age should address these limitations, especially with regards to population, diet and fecal microbiome analysis. Extending the timeframe of beyond 11 years of age would allow better exploration of the senior lifestage and associated changes, while including functional assessments, such as cognitive or mobility assessments, may allow identification of changes to the metabolome and lipidome which are predictive of functional outcomes and development of biomarkers for functional outcomes. It is clear from this work that using metabolomics and lipidomics to assess changes to circulating and excreted metabolites provided a more nuanced picture of metabolism with aging than clinical biochemistry markers alone, and the use of advanced analytical technologies can deepen our understanding of feline aging.

## 5 Conclusion

Aging in cats leads to a wide range of changes to plasma and fecal metabolites and lipids, characterized by two distinct metabolite and lipid trajectories; relatively consistent change over time, and a U-shaped response that mirrors the proposed life stages of cats. The totality of the metabolite and lipid responses are suggestive of mechanisms associated with aging similar to other species including inflammaging, mitochondrial dysfunction, alteration to lipid and amino acid turnover and oxidation, and possibly dysbiosis. Analysis of both plasma and feces enabled a wider perspective on the metabolic response to aging, including a potential role for the intestinal microbiome. Identification of these age-related metabolic changes provides potential biomarkers for assessing biological aging in felines, and the clear shifts observed between 6 and 8 years of age suggest that early intervention meant at mitigating some of these processes could help to support a healthy aging trajectory in cats.

## Data Availability

The raw data supporting the conclusions of this article can be made available by the authors for non-commercial purposes only.

## Supplementary material

Supplementary Figure 1 Principal component analysis by age **A)** plasma metabolomics, **B)** plasma lipidomics, **C)** faecal metabolomics, **D)** faecal lipidomics. QC = quality control sample for each sample matrix.

Supplementary Figure 2 Principal component analysis by analysis batch and type of diet fed. **A)** plasma metabolomics, **B)** plasma lipidomics, **C)** fecal metabolomics, **D)** fecal lipidomics. Batches: B1 to B5; types of diet: E - extruded, R - retorted, M - mixed. QC: Quality Controls.

Supplementary Figure 3 Fixed effects of top 10 individual metabolites from plasma metabolomics data. Predicted values are represented relative to year 1.

Supplementary Figure 4 Fixed effects of top 10 individual metabolites from plasma lipidomics data. Predicted values are represented relative to year 1. PE – Phosphatidylethanolamine, PC – Phosphatidylcholine, PI – Phosphatidylinositol, ST – Sterol.

Supplementary Figure 5 Fixed effects of top 10 individual metabolites from fecal metabolomics data. Predicted values are represented relative to year 1.

Supplementary Figure 6 Fixed effects of top 10 individual metabolites from fecal lipidomics data. Predicted values are represented relative to year 1. SM – Sphingomyelin, LPC – Lysophosphatidylcholine, TG – Triglyceride, CoQ – Coenzyme Q.

Supplementary Figure 7 Fixed effects of top 10 individual biochemical markers. Predicted values are represented relative to year 1.

Supplementary Datasheet 1 Mean (95 % CI) for parameters from the standard serum biochemistry panel measured in cats in this study (*n* = 12). Grey dashed lined indicate the reference range expected for each marker. One cat had abnormally high values for some clinical markers for the year seven plasma sample, likely due to hemolysis of the blood sample. This sample was removed from all clinical measures, so for year seven *n* = 11.

Supplementary Table 1 Number of samples for each analysis type by year.

Supplementary Table 2 RM-ASCA^+^ parameters.
