# Supplementary material for: Metabolomic and lipidomic signatures of aging in cats reveal shifts in energy metabolism and inflammation: an 11-year longitudinal study
Source: Front Vet Sci. 2026 Oct 21;13:1953163. doi: 10.3389/fvets.2026.1953163 (PMC13731272; doi:10.3389/fvets.2026.1953163)
Supplement: Supplementary file 1 [file Data_Sheet_1.PDF]

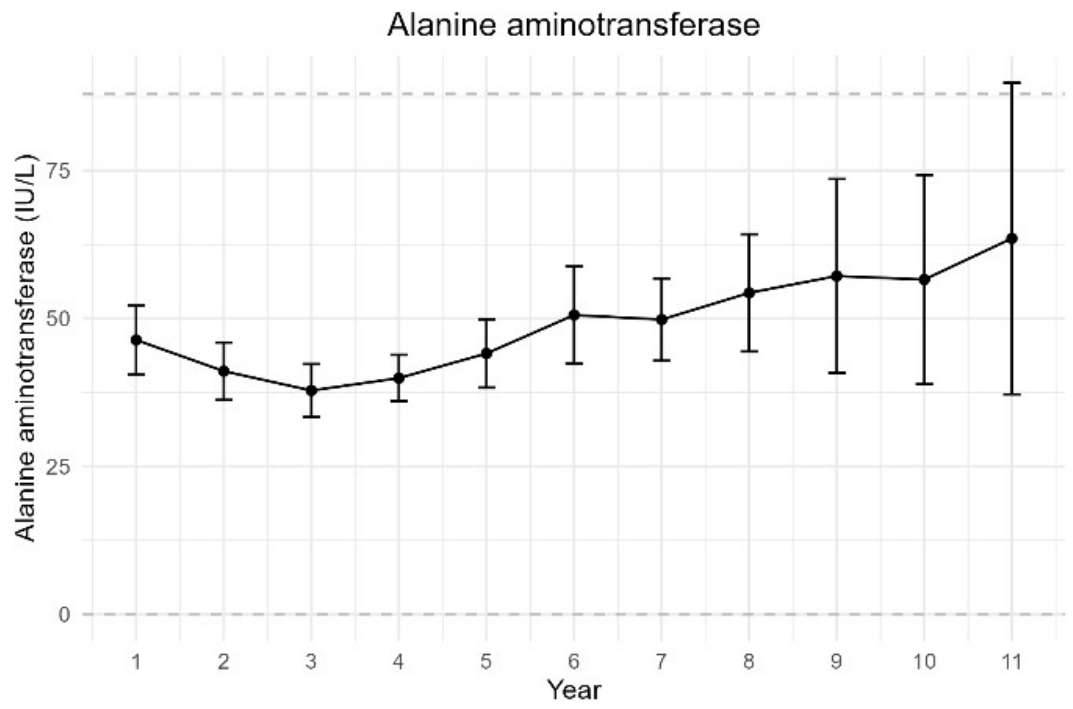
Alanine aminotransferase
Alanine aminotransferase (IU/L)
0
25
50
75
1 2 3 4 5 6 7 8 9 10 11
Year

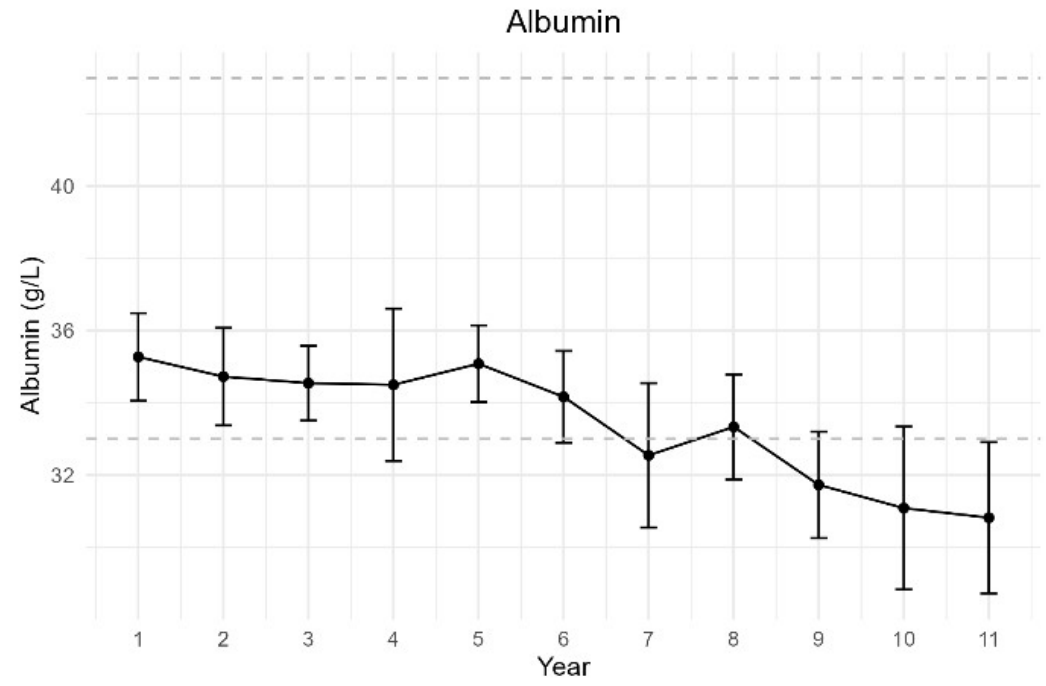
Albumin
Albumin (g/L)
32
36
40
1 2 3 4 5 6 7 8 9 10 11
Year

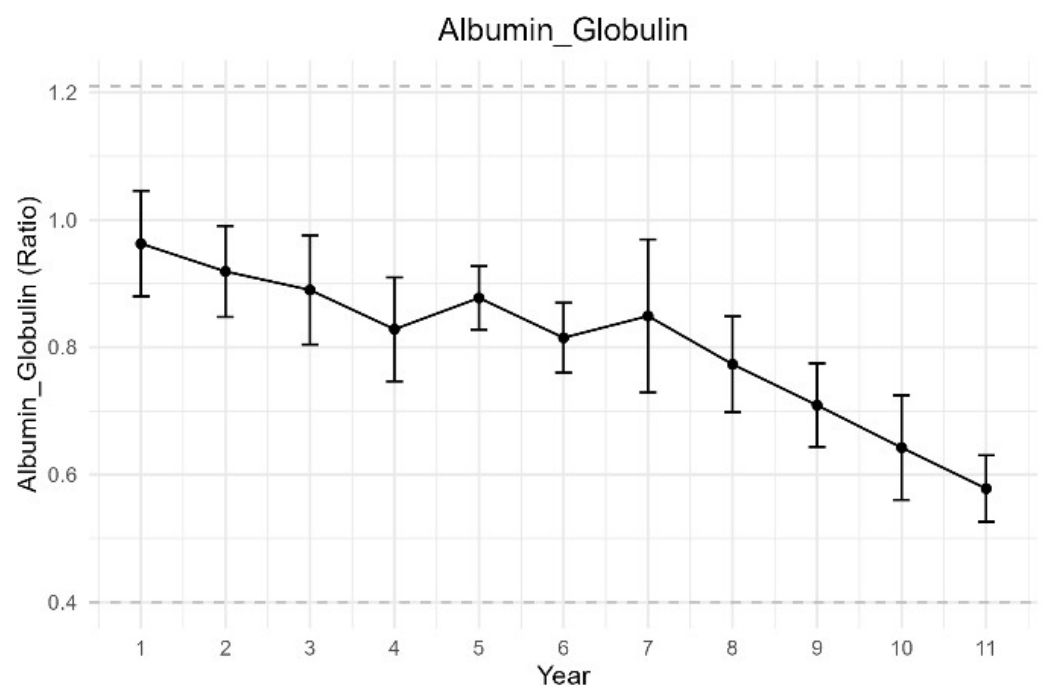
Albumin_Globulin
Albumin_Globulin (Ratio)
0.4
0.6
0.8
1.0
1.2
1 2 3 4 5 6 7 8 9 10 11
Year

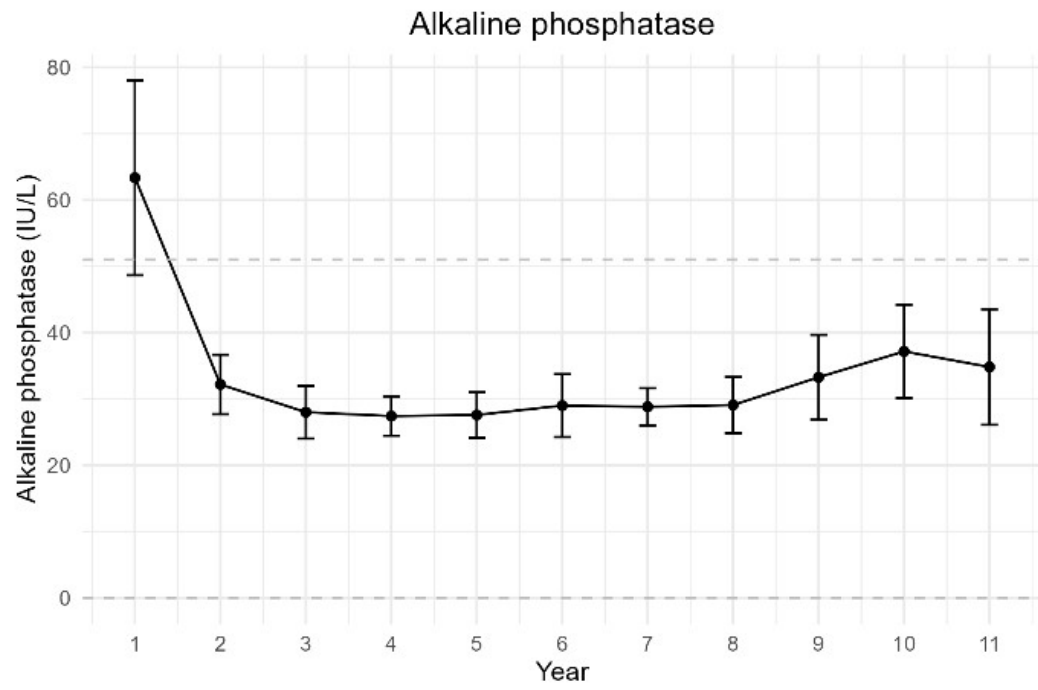
Alkaline phosphatase
Alkaline phosphatase (IU/L)
0
20
40
60
80
1 2 3 4 5 6 7 8 9 10 11
Year

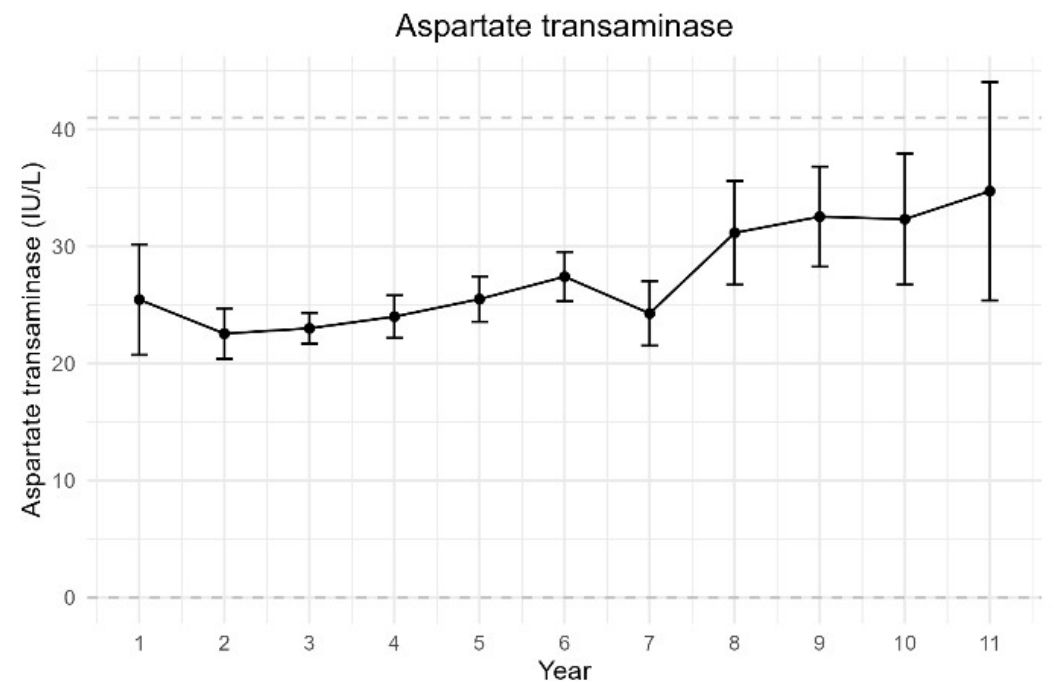
Aspartate transaminase
Aspartate transaminase (IU/L)
0
10
20
30
40
1 2 3 4 5 6 7 8 9 10 11
Year

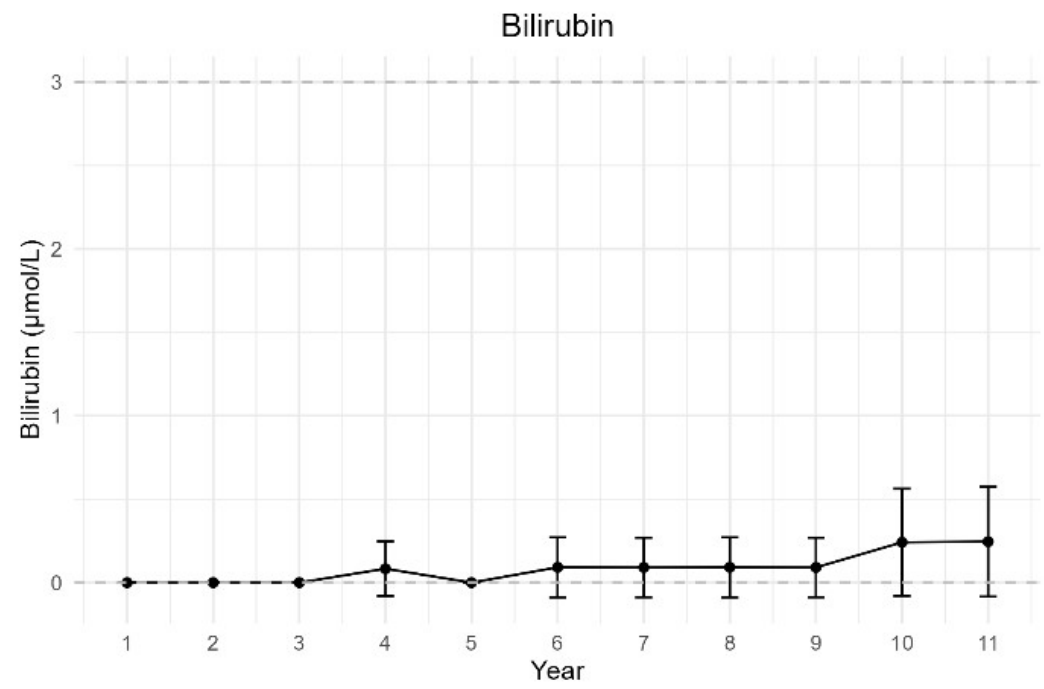
Bilirubin
Bilirubin (µmol/L)
0
1
2
3
1 2 3 4 5 6 7 8 9 10 11
Year

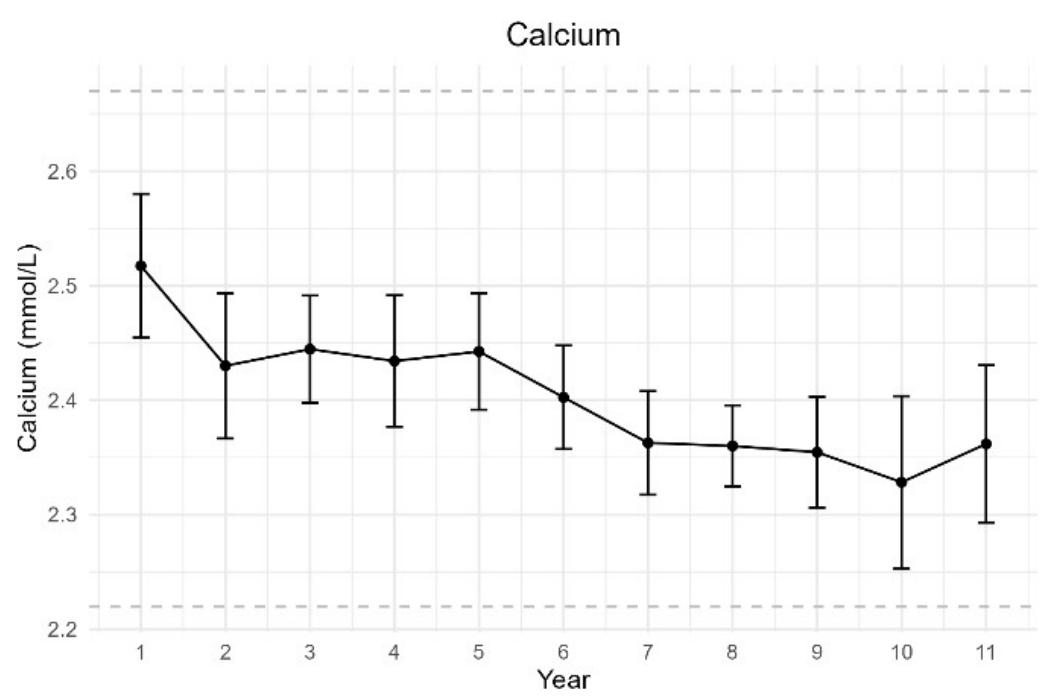
Calcium
Calcium (mmol/L)
2.2
2.3
2.4
2.5
2.6
1 2 3 4 5 6 7 8 9 10 11
Year

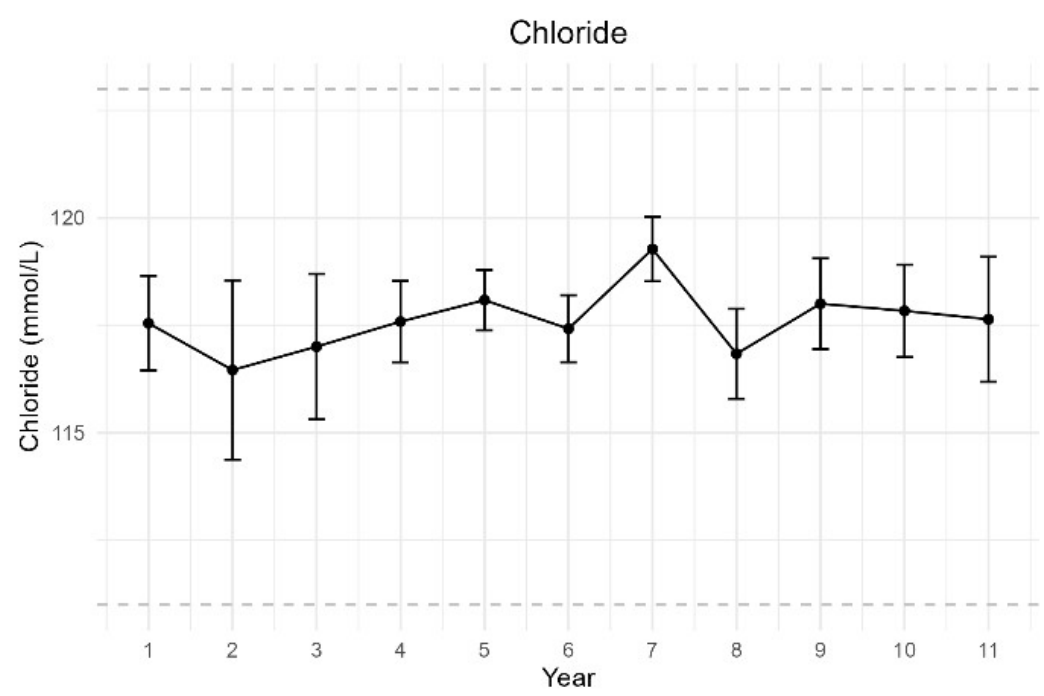
Chloride
Chloride (mmol/L)
115
120
1 2 3 4 5 6 7 8 9 10 11
Year

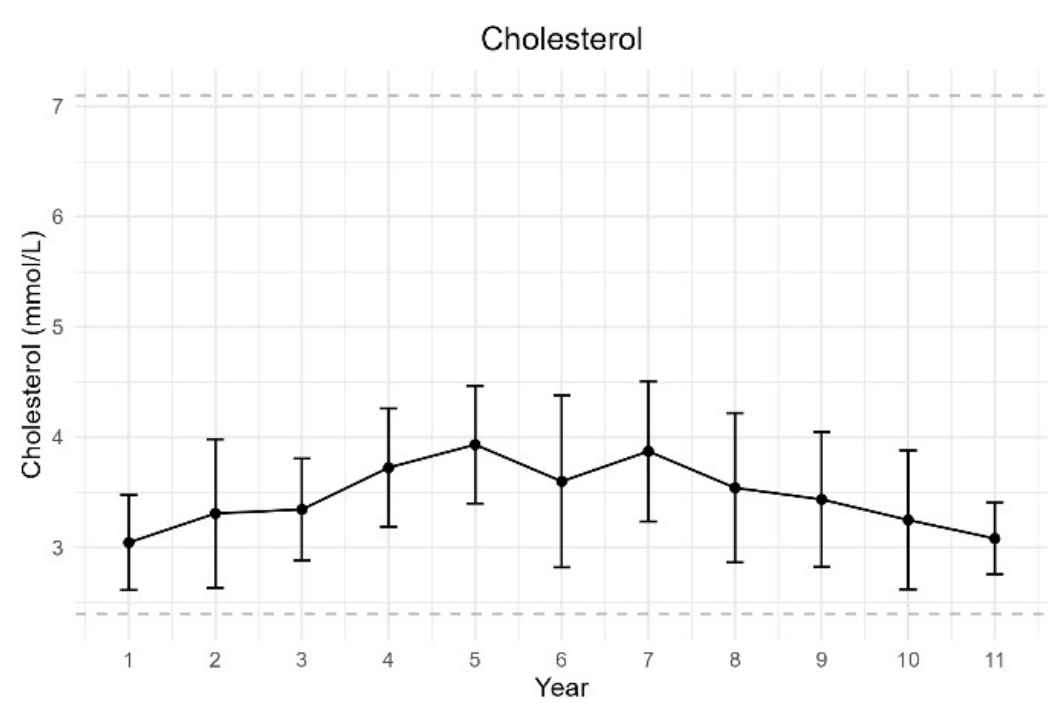
Cholesterol
Cholesterol (mmol/L)
3
4
5
6
7
1 2 3 4 5 6 7 8 9 10 11
Year

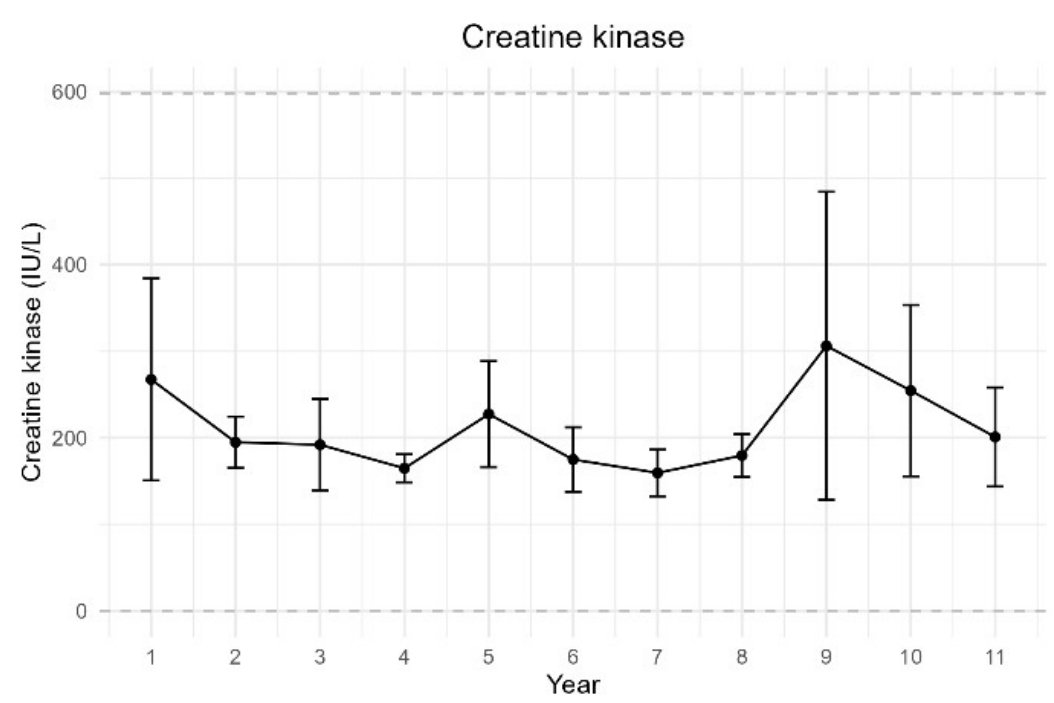
Creatine kinase
Creatine kinase (IU/L)
0
200
400
600
1 2 3 4 5 6 7 8 9 10 11
Year

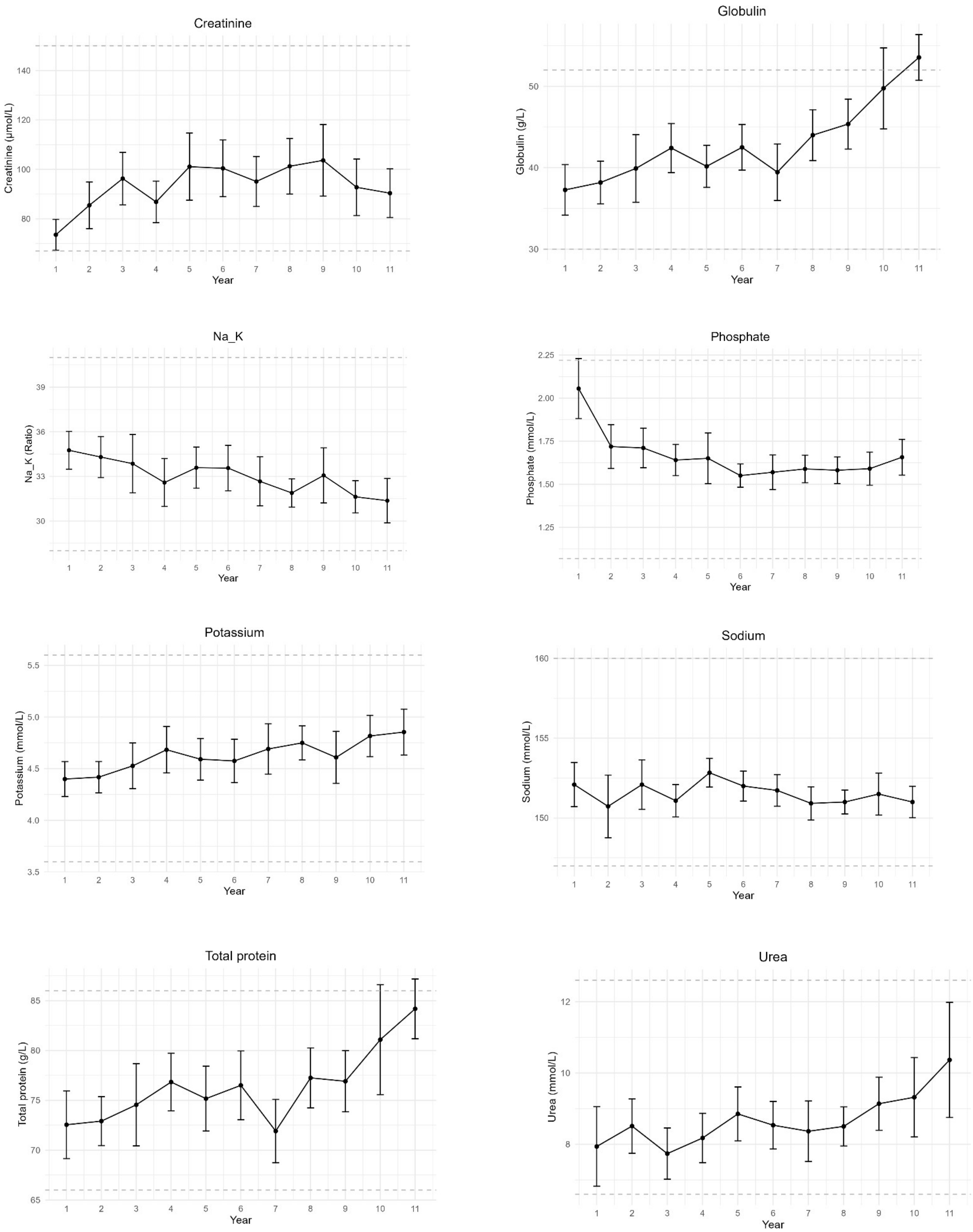

Creatinine
Creatinine (µmol/L)
80
100
120
140
1 2 3 4 5 6 7 8 9 10 11
Year
Globulin
Globulin (g/L)
30
40
50
1 2 3 4 5 6 7 8 9 10 11
Year
Na_K
Na_K (Ratio)
30
33
36
39
1 2 3 4 5 6 7 8 9 10 11
Year
Phosphate
Phosphate (mmol/L)
1.25
1.50
1.75
2.00
2.25
1 2 3 4 5 6 7 8 9 10 11
Year
Potassium
Potassium (mmol/L)
3.5
4.0
4.5
5.0
5.5
1 2 3 4 5 6 7 8 9 10 11
Year
Sodium
Sodium (mmol/L)
150
155
160
1 2 3 4 5 6 7 8 9 10 11
Year
Total protein
Total protein (g/L)
65
70
75
80
85
1 2 3 4 5 6 7 8 9 10 11
Year
Urea
Urea (mmol/L)
8
10
12
1 2 3 4 5 6 7 8 9 10 11
Year
